# Organoid cell fate dynamics in space and time

**DOI:** 10.1126/sciadv.add6480

**Published:** 2023-08-18

**Authors:** Xuan Zheng, Max A. Betjes, Pascal Ender, Yvonne J. Goos, Guizela Huelsz-Prince, Hans Clevers, Jeroen S. van Zon, Sander J. Tans

**Affiliations:** ^1^ AMOLF, Amsterdam, Netherlands.; ^2^ Oncode Institute, Hubrecht Institute, Royal Netherlands Academy of Arts and Sciences and University Medical Center, Uppsalalaan 8, Utrecht 3584 CT, Netherlands.; ^3^ Bionanoscience Department, Kavli Institute of Nanoscience Delft, Delft University of Technology, Delft, Netherlands.

## Abstract

Organoids are a major new tool to study tissue renewal. However, characterizing the underlying differentiation dynamics remains challenging. Here, we developed TypeTracker, which identifies cell fates by AI-enabled cell tracking and propagating end point fates back along the branched lineage trees. Cells that ultimately migrate to the villus commit to their new type early, when still deep inside the crypt, with important consequences: (i) Secretory cells commit before terminal division, with secretory fates emerging symmetrically in sister cells. (ii) Different secretory types descend from distinct stem cell lineages rather than an omnipotent secretory progenitor. (iii) The ratio between secretory and absorptive cells is strongly affected by proliferation after commitment. (iv) Spatial patterning occurs after commitment through type-dependent cell rearrangements. This “commit-then-sort” model contrasts with the conventional conveyor belt picture, where cells differentiate by moving up the crypt-villus axis and hence raises new questions about the underlying commitment and sorting mechanisms.

## Teaser

Tracking all cell types in intestinal organoids reveals a commit-then-sort model that upends current differentiation models.

## INTRODUCTION

The past decade has brought groundbreaking advances in organoid technology, which enabled many new approaches to studying organ development, self-renewal, and pathology (*1*–*5*). The benefit of organoids lies in their ability to in vitro generate the diverse cell types and three-dimensional (3D) organization that characterize in vivo organ tissue. Key features of the cellular architecture have been established using immunostaining, fluorescence microscopy, and single-cell RNA sequencing (*6*–*10*). However, these methods do not reveal the dynamics that are critical to the underlying cellular reorganization and differentiation, as exemplified, for instance, by the rapidly renewing intestine. Lineage tracing (*11*–*14*) can visualize the offspring of some cells but not when or where cells adopt a new fate nor their movements or lineage trees. In parallel, important advances have been realized in 3D time-lapse microscopy. Confocal and light-sheet imaging can visualize the dynamics of cell proliferation and migration (*10*, *15*–*18*), while machine learning methods can track cells in time (*17*, *19*–*21*). However, this approach has been of limited use in studying the dynamics of differentiation. While fluorescent protein markers can, in principle, be used to identify cell types (*22*–*24*), the many involved types (over five in intestinal organoids) (*9*) and the fluorescent labeling of nuclei required for tracking present major challenges in terms of phototoxicity, genetic engineering, and wavelength overlap.

Hence, the dynamic basis of cellular organization has not been resolved for any organoid system (*25*). In intestinal organoids, stem cells are thought to differentiate when moving into the transit-amplifying (TA) region higher up in the crypt, driven by proliferation at the crypt bottom, upon which they stop proliferating and continue upward to the villus region, where most differentiated cells reside and are ultimately shed into the lumen (*26*–*29*). Cues are provided by Wingless-related integration site (WNT) activity that decreases along the crypt-villus axis and Notch inhibition between secretory and absorptive types that regulate their relative abundance (*30*–*32*). This “conveyor belt” model is appealing because it can naturally explain how different types are dynamically organized in space: Cells driven toward the differentiated villus region are triggered to differentiate, while cells remaining in the crypt preserve their stem identity. However, with dynamic data lacking, it remains unclear whether this principle holds and whether other mechanisms contribute. Addressing these issues requires the characterization of all cells within key regions.

**Fig. 1.**
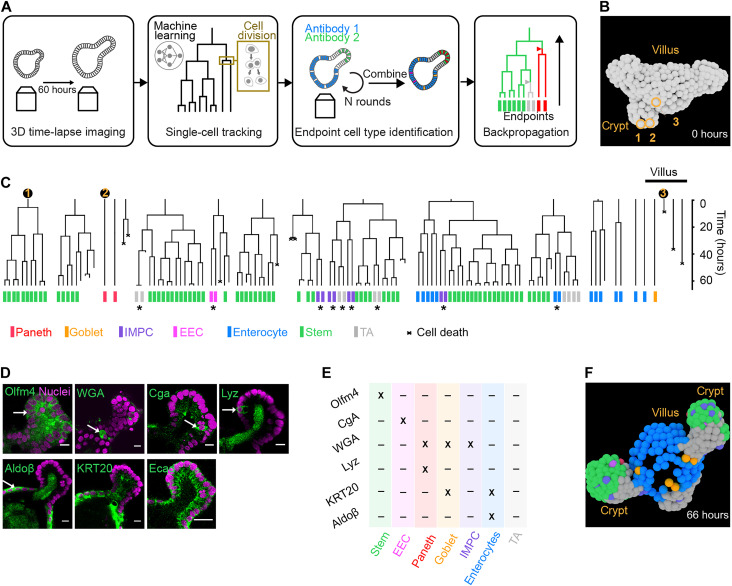
TypeTracker method overview and primary data. (**A**) Method workflow. Step 1: Image several organoids in 3D for 60 hours. Step 2: Track all cell trees using convolutional neural network. Each cell is uniquely identified from frame to frame, yielding a lineage (black line). Division events give a fork-like structure. Step 3: Identify cell type at movie end point using multiple rounds of antibody staining. Step 4: Map cell types onto lineage tree and infer historical cell types including type transitions (triangles). (**B**) Reconstructed cell positions at the start of the lineage tree. (**C**) Example lineage trees and their mapped end point cell types. Numbers, lineages of three cells seen in (B); stars, end point sister pairs of same type but of different type than cousins, suggesting type transition in mother cell. (**D**) Nuclear marker (magenta) and antibody or dye staining (green). Olfm4, olfactomedin 4; WGA, wheat germ agglutinin; Cga, chromogranin A; Lyz, lysozyme; Aldoβ, aldolase β; KRT20, cytokeratin 20; Ecad, E-cadherin. Scale bar, 10 μm, except for the Ecad panel (20 μm). (**E**) Cell type identification table. (**F**) Reconstructed cell positions and type at end of the lineage tree (C) and 66 hours after the start (B).

We, therefore, developed a method that integrates type identification and tracking (TypeTracker). It works by propagating cell types back in time along lineage trees using machine learning–based analysis of 3D video microscopy, multiplexed antibody staining of end point cells, and a rule-based backpropagation protocol (Fig. 1A). The resulting multidimensional data identifies the moment and location where cells differentiate, their movements and genealogical relations to other cells, and the neighbors that they interacted with. It can be used for any organoid with proliferating and differentiating cells and requires only confocal microscopy, immunostaining, and data integration software.

Application to mouse intestinal organoids (Fig. 1B) (*1*) revealed an unexpected principle for the interplay between differentiation and spatial organization. Type commitment occurred notably early: well before the differentiating cell lineages ceased to proliferate and moved upward to the villus region, at a moment when the cells still occupied the same region as their stem cell relatives that continued to retain the stem identity and remained deep inside the crypt. This early commitment had several notable features. Secretory fate emerged typically with identical types in pairs of sister cells, indicating that their mothers had committed already and contrasting with the notion that committed cells arrest their cell cycle (*33*–*35*). While cells of different secretory types were observed, they were not closely related and rather emerged from distinct stem cell lineages. This finding challenges the existence of omnipotent secretory precursors that produce progeny of different secretory types (*33*, *34*, *36*–*39*). Absorptive lineages are committed even longer before cell cycle arrest (over five divisions), providing an explanation for the high abundance of absorptive over secretory types. In contrast with current models (*26*, *40*, *41*), the stereotypical positioning of cell types was achieved through spatial segregation after commitment and secretory sister cells separating more rapidly than neighboring cells of different types. Given the conserved nature of the underlying pathways such as WNT and Notch, this “commit-then-sort” mechanism may well be widely relevant. Our TypeTracker approach can be used broadly to elucidate the dynamic differentiation programs of organoid systems and how they are affected by mutations or external conditions and can be integrated with live cell monitoring of key cell biological processes.

## RESULTS

### Overview of TypeTracker

Our method consists of four main steps (Fig. 1A). First, movies of organoids growing in basement membrane extract (BME) gel are recorded by 3D confocal microscopy (fig. S1, A to C). We focus on intestinal organoids and image complete organoids or their crypt protrusions every 12 min for 60 hours using a histone 2B (H2B)–mCherry marker to detect cell nuclei (fig. S1D). Second, we use a convolutional neural network (*19*) to track the nuclei in space and time across several generations (Fig. 1B and fig. S1, E and F) and visualize their genealogical relations using lineage trees (Fig. 1C and figs. S2 and S3). The tracked organoids had different sizes, but all showed similar cell cycle durations. Consistently, proliferative lineages, which display up to seven divisions, are mostly found at the crypt bottom, while nondividing cells are abundant in the villus-like region of the organoid. Third, we perform multiple rounds of antibody staining to determine the cell types at the movie end point, as detailed below (Fig. 1, D and E). Hence, we identify the main types found in the intestine: stem, enteroendocrine, Paneth, goblet, enterocyte, TA, and an immature mucus-producing type. Fourth, we infer the cell types at earlier time points, starting at the end point of the lineage tree and progressively moving back along each branch to the beginning, using a set of rules that we describe in detail below. Hence, one obtains virtual organoids that present the spatial location, division events, movements, and type of all cells (Fig. 1F). The completeness of this method, which identifies all cells in a region of interest and associated lineage trees, enables our backpropagation approach and analysis of spatiotemporal correlations.

### Cell type identification

We developed an approach to map cell type data, as obtained by several rounds of antibody staining, onto the end point of the movie and lineage trees (Fig. 1, D and E). This method includes washing protocols that minimize organoid deformations, antibody stripping between rounds, and a minimum-cost flow solver algorithm (*42*) combined with manual correction to link cells in the staining images to the cells at the movie end point. Stem cells were identified by olfactomedin 4 (Olfm4) (*43*); enterocytes, by aldolase β (Aldoβ) (*10*); and enteroendocrine cells (EECs), by chromogranin A (Cga) (*44*). Consistently, Olfm4^+^ cells were found only at the crypt bottom; Cga^+^ cells, in both the crypt and villus region; and Aldoβ^+^ cells, exclusively in the villus region (Fig. 1F). Stem cells and EECs did not stain for any of the other markers used, while enterocytes also stained for cytokeratin 20 (KRT20) (*45*), as expected. Wheat germ agglutinin (WGA), which stains intestinal mucus (*46*, *47*), labeled cells in the crypt and villus regions. A subset of these were also positive for lysozyme (Lyz) and thus identified as Paneth cells (*48*), while another subset expressed KRT20 and was identified as goblet cells (*45*). Consistently, the former often contained granules that are typical of Paneth cells, while the latter had a cup-shaped morphology that characterizes goblet cells (fig. S4). A remaining subset of WGA^+^ cells expressed neither Lyz nor KRT20. Evidence provided below suggests that these are immature Paneth and goblet cells, which we thus refer to as immature mucus-producing cells (IMPCs). We identified a population of cells within the crypt that did not stain for any of our markers, which are identified as TA cells on the basis of evidence discussed below.

### Sister cells adopt the same type

**Fig. 2.**
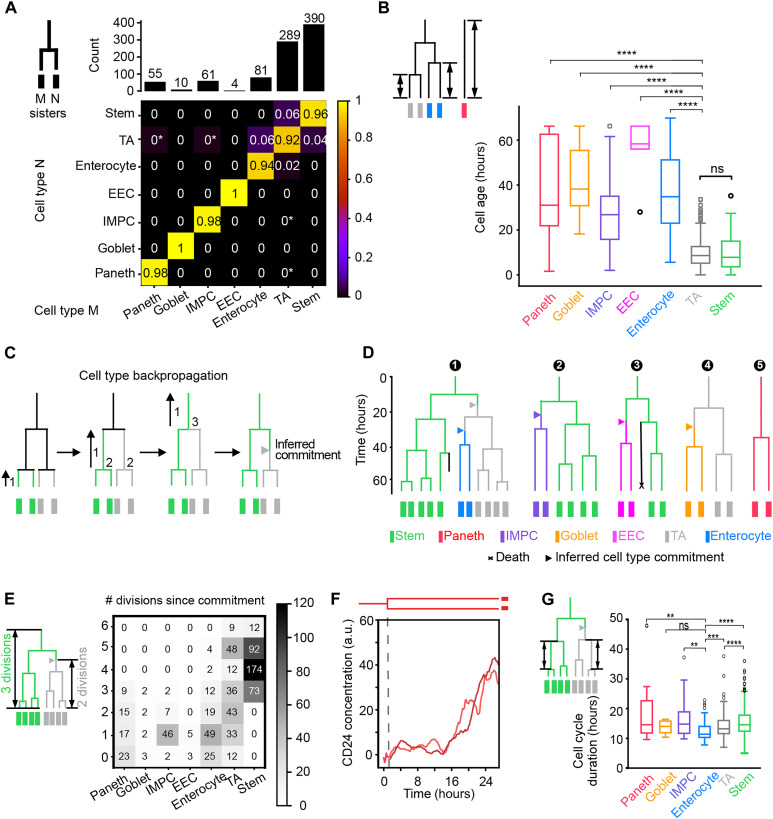
Differentiation pathways and cell type commitment. (**A**) 2D histogram indicating the two cell types of sisters at the movie end point, as determined using antibody staining. Shown is the number of times a cell type combination was observed divided by the total of each column, indicated on top. Star: low frequency resulting from a single observation. The dominant diagonal shows that sisters typically have the same type. (**B**) Age of stained cells, quantified as the time since the last division or since movie start for lineages without division. ****P ≤ 0.0001; not significant (ns), *P* > 0.05. TA cells show the same age as stem cells, indicating their proliferative nature. Consistently, the differentiated cells (EECs, enterocytes, Paneth, and goblet cells) are older. (**C**) Cell type backpropagation rules. 1: Working backward along each lineage, the inferred type is unchanged when not encountering branch points (divisions). 2: For branch points, if both daughters are the same type, then the mother is assigned that type. 3: If only one daughter is a stem cell, then the mother is assigned as a stem cell. 4 (not shown): If only one daughter is a TA cell and the other is not a stem cell, the mother is assigned as a TA cell. In cases 3 and 4, a type transition is inferred in one daughter (triangle). (**D**) Example lineage trees as determined by TypeTracker. (**E**) Number of observed divisions for each cell type. (**F**) Cell division generating two Paneth daughters showing similar profiles of increasing CD24 signals. (**G**) Cell cycle duration for different cell types. Cell cycle durations were notably similar between types, while enterocytes exhibited lower mean cell cycle durations than TA cells, stem cells, and the secretory cell types. All data in this figure were from nine organoids. a.u., arbitrary units. **P ≤ 0.01 and ***P ≤ 0.001.

Prominent in our data is that end point types generally came in pairs, with sister cells consistently displaying the same fate (Fig. 1C). Statistical analysis showed that 97% of all end point sister (*N* = 869 sister pairs in 9 organoids) were of the same type. All types, including secretory types, exhibited this symmetry, as evidenced by the dominant diagonal in the sister-sister cell type histogram (Fig. 2A). This consistent type symmetry between sisters is notable. Intestinal stem cells are proposed to exit from their cell cycle when committing to a secretory fate (*33*, *34*), which would rather yield asymmetry, as one sister may then adopt a secretory fate that the other does not. External cues can, in principle, produce a local environment that is similar for the two sister cells and hence drive them to the same fate. However, a priori, there is then no reason why only sisters should be affected, as also nonsister cells like cousins can be neighbors. The fates of many sister pairs were different from their direct cousins (Fig. 1C, stars). This showed that the underlying type transitions did affect sisters specifically and occurred during the observed growth period of the lineage. These data indicated that the type transitions rather occurred in the mother (or earlier generations), which subsequently divided to produce two daughters of the same type. Larger subtrees, which, for instance, showed two or more sister pairs of the same type [Fig. 1C (blue enterocyte subtree) and figs. S2 and S3], were consistent with divisions occurring after type commitment and hence producing cells of the same type.

We similarly observed symmetry between goblet sisters in conditions that enriched for goblet cells [Inhibitors of Wnt production compound (IWP2) and Gamma secretase inhibitor (DAPT)] (*22*, *49*). For those goblet lineages that showed a division, the sister cell also adopted the goblet fate (fig. S5). We also obtained more evidence for symmetry between Paneth sister cells (see below) and EECs: A fluorescent reporter of neurogenin 3 that marks EEC cells (*22*) was found to increase simultaneously in two nearby cells (fig. S6).

### Dependence of cell age on differentiation state

The age of the cells at the movie end point was defined as the time since the last observed division (Fig. 2B). Notably, the TA cells showed a statistically identical age distribution as the stem cells, in line with the notion that they are highly proliferative (*34*, *40*, *50*). EECs, enterocytes, Paneth, and goblet cells also showed a broad distribution but with an average age that was substantially larger than the stem and TA cells, consistent with their terminally differentiated nature. The ages of these terminally differentiated cells were rarely below 20 hours (Fig. 2B), suggesting that the involved markers (Cga, Aldoβ, Lyz, and KRT20) took a substantial amount of time to become observable.

### Cell type backpropagation

To infer the cell type along the lineages of the family tree, we used the lineage tree topology and the above observations (Fig. 2, A and B). Starting at the lineage end points, we propagated the end point types back in time using this process: (1) From one time point to the previous time point, the type is initially assigned as unchanged if branch points (divisions) are not traversed but may later be updated when a cell type transition is identified. (2) At branch points, the two involved daughters are considered: If both display the same (inferred) type, then the mother is assigned as that type, as discussed above (Fig. 2A). (3) If one daughter is a stem cell and the other is not, then the mother is assigned as a stem cell on the basis of the notion that stem cells are generated from stem cells. (4) If one daughter is a TA cell and the other is not, nor a stem cell, then the mother is assigned as a TA cell. This rule is based on the TA cells being proliferative like stem cells (Fig. 2B), which makes the alternative (the mother has the identity of the non-TA daughter) unlikely. In the last two cases (3 and 4), a type transition is inferred in one of the daughters. We mark the latter with an arrow halfway the cell cycle (Fig. 2C). These rules allowed the backpropagation of complete trees from the end to the beginning (Fig. 2D and fig. S7).

Next, we estimated stemness by quantifying the fluorescence intensity of Leucine-rich repeat-containing G-protein coupled receptor 5 (Lgr5)-GFP (green fluorescent protein), a well-known stem cell marker (*51*), to further test our method. Phototoxicity, which is stronger for GFP than for mCherry, limits the imaging frequency and duration. GFP at the movie end point correlated with measured Olfm4 staining intensities (fig. S8, A and B). Backpropagation-inferred stem cells showed higher GFP expression, while inferred enterocytes or TA and goblet cells showed lower GFP (fig. S8, C and D). Notably, lineages inferred to go from stem to TA type showed decreasing GFP, and those decreases were established in the mother cell, identified as the committing cell by the TypeTracker method (fig. S8E), thus supporting the backpropagation approach.

Paneth cells also showed consistency between backpropagated type and real-time visualization, by identifying the granules that characterize Paneth cell (fig. S9A). The latter also showed Paneth cell division (fig. S9A). Next, we performed time-lapse imaging with a Paneth cell marker (CD24; fig. S9B). Most CD24^+^ cells did not show divisions, as expected for terminally differentiated Paneth cells. We did see cells that divided and then became CD24^+^. Notably, the corresponding sister cell then also showed the CD24 signal increasing in the same manner (Fig. 2F and fig. S9, C and D). These data are consistent with the mother committing to the Paneth fate and CD24 expression following later in the daughters. These findings highlight the advantage of our method when markers are visible only long after commitment and hence not informing on early differentiation events. Note that limitations of our method are described in the discussion.

### Differentiation pathways

The observed differentiation pathways indicated notable features (Fig. 2D and fig. S7). Trees typically displayed one to three cell type transitions, thus yielding subtrees of the new type, while the old type was maintained in another subtree, and some lineages showed two consecutive type transitions. For instance, a stem cell tree was first shown to spawn a TA subtree, which, in turn, generated an enterocyte subtree (Fig. 2D, tree 1). This order, in which TA is an intermediate type between stem and terminally differentiated types (*26*), is consistent with the sister-sister cell type histogram: Besides the dominant diagonal, low-frequency off-diagonal entries indicated stem-TA sister pairs and a few pairs of one TA and one terminally differentiated type but never sister pairs showing two different terminally differentiated types (Fig. 2A). The latter would be in line with models where stem cells first differentiate into secretory precursors, which, in turn, can generate different terminally differentiated types (*33*, *34*, *36*–*39*). To further probe this notion, we extracted the largest possible subtrees from our data that contained two types and found that none had two different secretory types nor one secretory and one absorptive type. Instead, they typically combined TA and either stem (52.2%) or a terminally differentiated state (31.4%; fig. S10), consistent with the sister relations (Fig. 2A). Among these subtrees, we also found cases (16.4%) that combined stem and terminally differentiated states (Fig. 2D, tree 2 and 3), suggesting that these lineages have a negligible TA role.

### Division rather than differentiation rates control enterocyte abundance

Cell type transitions to enterocytes were less frequent than those to the secretory types combined (about 1.5-fold), even as enterocytes outnumbered secretory cells. To investigate this issue, we quantified the number of consecutive divisions in each differentiation state (Fig. 2E). The EEC, goblet, and IMPC secretory states mostly showed one division, sometimes two. In contrast with common thinking (*33*, *34*), we found Paneth cells to divide as well (Fig. 2E). The inferred Paneth cell divisions were confirmed by the continuous presence of granules that are specific to Paneth cells and are observable without labeling (fig. S9A). Next, we quantified the cell cycle duration for each cell type. Notably, these complete cell cycles were similarly long for all types including secretory and stem types (Fig. 2G). The lineages were observed to stop dividing subsequently, consistent with a cell cycle exit.

To further probe proliferation of secretory cells, we costained with the Paneth marker Lyz and the proliferation marker Ki67. Consistently, 33% of the Lyz^+^ cells were also Ki67^+^ (fig. S11A). We also studied cells with a Neurogenin 3 label that marks EEC cells and found that a small fraction was Ki67^+^, consistent with committed EEC secretory cells capable of dividing (fig. S11C).

Enterocyte lineages displayed significantly more divisions than secretory cells. The former reached to up to five divisions, not far off from the six divisions seen for stem and TA states (Fig. 2E). Hence, after specification, absorptive lineages generated substantially more cells than secretory lineages, which thus offset their comparatively low transition rate. This finding is consistent with recent data in which absorptive lineages showed larger clone sizes than secretory lineages (*13*, *14*). Enterocytes were even found to exhibit the lowest mean cell cycle duration (12.5 hours), significantly lower than for both stem (15.5 hours) and TA cells (14.1 hours; Fig. 2G). Overall, these results indicate that relative abundance is controlled by the cell lineage dynamics, particularly the number of divisions after specification.

### The spatiotemporal differentiation program

**Fig. 3.**
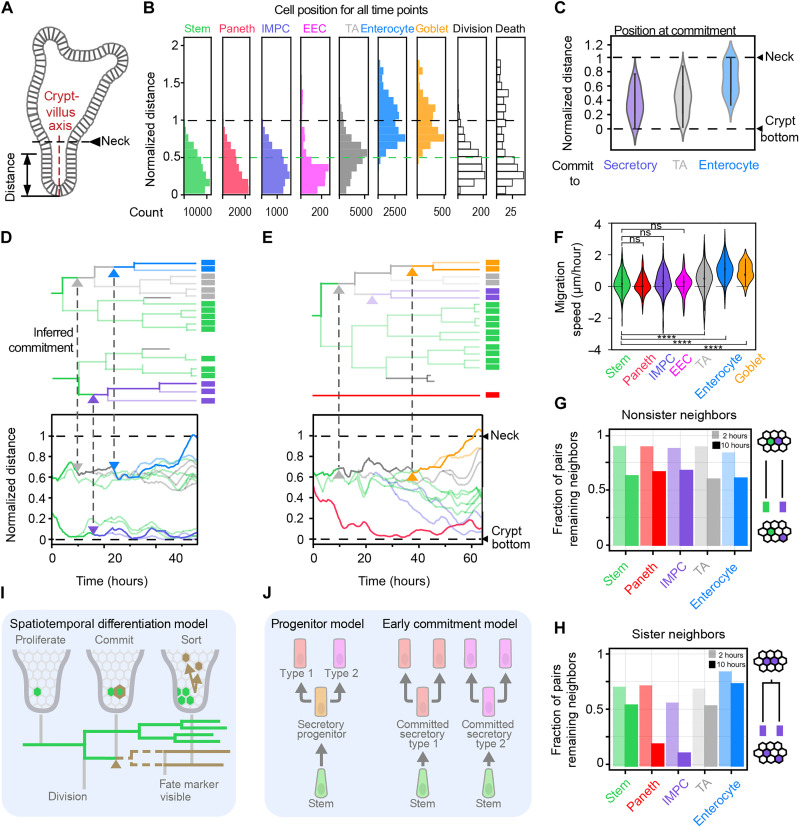
Spatiotemporal differentiation program. (**A**) Organoid schematic diagram. (**B**) Inferred cell types mapped along the normalized crypt-villus axis. (**C**) Position of cells when they commit to a new type. (**D** and **E**) Lineage trees mapped in space along the crypt-villus axis. Bottom trees: Transition to IMPC and position of Paneth cell deep in the crypt surrounded by stem cells. Top trees: Transitions to enterocytes or goblet cells higher in the crypt but close to their stem cell relatives, followed by their movement to the villus region, rather than the other way around. (**F**) Cell migration speed, as quantified by the migration distance along the crypt-villus axis after commitment or movie start, divided by the migration time. ****P ≤ 0.0001; not significant (ns), *P* > 0.05. (**G**) Cell separation for one cell of the specified type and one cell of any type. Displayed is the fraction of pairs that remain neighbors in 2 or 10 hours. Most neighbors remained neighbors even after 10 hours, independently of cell type. (**H**) Cell separation of sisters of the same type. Sisters are shown to separate faster than nonsisters (G), particularly sister Paneth cells and IMPCs. (**I**) Commit-then-sort model. Cells commit early (to unspecified fate, brown triangle), when still nearby their stem cell relatives, and after which they can still undergo division. Tested fate markers rise in a detectable manner after a certain time only. Spatial patterning occurs after commitment by type-dependent rearrangements. (**J**) Differentiation pathway models. In the first, stem cells first become an omnipotent secretory progenitor that can divide and produce different secretory types. In the second, cells commit early to different secretory types and produce cells of the same type when they divide. Our dynamic data are consistent with the latter model.

Access to spatial dynamics is a key benefit of our approach. The positions of inferred cell types mapped along the crypt-villus axis as expected, with stem and Paneth most toward the bottom, followed by IMPC, EEC, TA, enterocyte, and goblet (Fig. 3, A and B, and fig. S12A) (*26*, *28*) . In addition, we can now map the point of commitment onto the spatial organoid structure. Commitments to secretory types were broadly distributed along the crypt-villus axis but typically occurred deeper in the crypt than absorptive commitments (Fig. 3C). The latter also distributed broadly and were positioned well within the crypt. Notably, when we mapped full lineage trees along the crypt-villus axis, we found that these committing cells were still in the same approximate spatial region as their close relatives that maintained the stem fate (Fig. 3, D and E, and fig. S12, B and C). Spatial separation from these stem cell relatives did occur after committing (Fig. 3, D and E). This separation was most evident for lineages destined for the villus region (enterocytes and goblet cells), with the stem lineages that they arose from remaining confined to the crypt [Fig. 3, D and E (top trees)]. Such separation from relatives was not always observed, specifically for cells committing to a secretory type (IMPC) near the crypt bottom and remaining there [Fig. 3D (bottom tree) and Fig. 3E (top tree)]. Consistently, the mean migration speed is highest for enterocytes and goblet cells and lowest for secretory fates like Paneth cells and IMPCs (Fig. 3F).

### Promotion of sister separation

Secretory types were typically born as sisters (Fig. 2A) and, hence, as neighbors. We wondered how this finding relates to the observation of a single Paneth being surrounded by stem cells (*1*, *52*). In our data, most neighbors that were not sisters (and hence can be of different type) were still neighbors after 2 hours (85%) and 10 hours (57%), independently of cell type (Fig. 3, G and H, and fig. S12D). Neighbors that were sisters, which almost always were of the same type, separated more frequently after 2 hours (71% remains neighbors), likely because of arrangements directly following divisions (*53*, *54*). Unexpectedly, Paneth and IMPC sisters showed even stronger separation over longer time scales and more than the other types, with less than 20% still neighbors after 10 hours (Fig. 3I). Sisters, and in particular of these secretory types, thus appear to rearrange more strongly to achieve interspersion. These findings show that active cell separation after fate commitment contributes to cell type patterning in the epithelium.

## DISCUSSION

Before discussing the findings, we review the limitations of our approach. First, transient fates like stem and TA present early in the tree may be missed, as their type may not be maintained until the end point (fig. S13, A and B). This issue is exacerbated by cell death events (crosses), as aborted lineages cannot propagate type information back in time (fig. S13, A and C), and partly mitigated by the fact that stem and TA types are proliferative and hence generate multiple branches. Second, if cells would show reversible transitions back to the stem or TA type, then not all of these events may be detected, again owing to their transient nature. Third, used antibodies may not identify immature types, which have committed briefly before the end of the movie. This issue affects marker-based methods more generally. Our data suggest lengthy type maturation, as evidenced by the dominant sister symmetry (Fig. 2A), high age of differentiated cells at movie end point (Fig. 2B), and live-cell marker data (Fig. 2F and fig. S6). Our method offers advantages in overcoming this limitations by detecting early commitment using lineage relations even when the phenotype is not detectably expressed yet.

Our data showed many features that are consistent with existing models of intestinal organoid renewal, including the presence of stem cell and TA zones, expected positions of differentiated cell types, and an effective migration along the crypt-villus axis driven by proliferation in the crypt bottom (Fig. 3, A and D to F) (*26*, *28*, *41*). The temporal order of events that our dynamic observation addresses was notable, however. Specifically, we found cells committing to a new type when still positioned in the same area as their stem cell relatives, well before their separation into the cell lineages that move toward the villus region and those that remain in the crypt (Fig. 3, C to E and I). The opposite was not observed: cells that first move away from their stem cell relatives and then differentiate when they have moved upward to the villus region, as in the classic conveyor belt model. We found commitment even deeper in the crypt for secretory types such as Paneth cells that ended up at the crypt bottom, thus not requiring the proposed downward migration after commitment (*26*, *55*). This observation agrees with a recent mouse study reporting that both Paneth cells and EECs arose directly from stem cells at the +4/+5 positions in the intestinal crypt (*54*). Another notable feature was the rapid separation of neighboring secretory sisters (Fig. 3, G to I), showing how local cell rearrangement can contribute to spatial cell type patterning. Such rearrangements may explain observed nonneighboring positions of clonal cells in the mouse intestine (*56*). Overall, our data indicated a picture in which cells first commit and subsequently spatially sort and reorganize (Fig. 3I).

This early commitment picture has other consequences. One example is the highly symmetric adoption of identical fates by sister cells, including those with secretory fates (Fig. 2A). It has been proposed that secretory differentiation occurs through an omnipotent secretory precursor that can generate all types of secretory cells (Fig. 3J) (*33*, *34*, *36*–*39*). Our early commitment findings suggest that this omnipotent secretory state, if it exists, occurs only briefly and is exclusively restricted to the mother cell, as different secretory fates are not seen among the mother’s offspring (Fig. 3J). This conclusion was supported by the more distant relatives: Subtrees containing two types at most never contained two different secretory types (fig. S10). An omnipotent secretory state was also questioned by a recent quantification of gene expression versus pseudotime, which indicated differentiation via lineage-specific unipotent transitions (*54*).

Our results indicate that type commitment occurs in mother cells that still undergo one or more divisions and transmit this type to its descendants (Fig. 3J). These findings contrast with the idea that cells exit their cell cycle upon committing to a secretory fate (*33*–*35*) and suggest that division may be important to completing differentiation (*57*). Live cell secretory markers (CD24 and Neurogenin 3) were not visibly expressed in the mother and, rather, rose simultaneously in the two daughters, in line with a committing mother (figs. S6 and S9). These data may also help explain why previous work suggested that committed secretory cells are nonproliferative (*33*–*35*). Reported clonal expansion of goblet cells after fate commitment in mouse colon is consistent with our findings (*13*). Notch signaling between neighbors is proposed to restrict secretory fate to a single cell that is surrounded by nonsecretory cells (*58*). Our observation of neighboring sisters with the same secretory fate (Fig. 3, H and I) implies that Notch signaling affects cell fate early and transiently, likely in the mothers of secretory cells.

We note several differences between organoid and in vivo development. Organoid crypts and villi lack the supporting mesenchymal tissue and, hence, the associated space constraints and range of molecular signals. In vivo, the mesenchyme can maintain stem cells in the absence of the Paneth cells (*59*–*63*), suggesting a redundancy in Wnt signaling that can affect aspects such as the size of the stem cell zone. In addition, organoids have an enclosed lumen that accumulates debris over time, which limits the observation time and could affect cellular metabolism. The ratio of differentiated and stem cells and the ratio of absorptive and secretory cells may also not directly compare between organoids and in vivo (*64*), although our data can show how increased divisions after enterocyte specification can be exploited to control their relative abundance over secretory cells (Fig. 2E). Last, organoids lack many other factors, including pathogens and interacting immune cells, which can promote the rare tuft and M-fold cells.

Our method can be applied broadly to study the spatiotemporal differentiation programs of organoid systems and how they are affected by external conditions such as metabolic compounds or interleukins, interacting cell types including bacteria and immune cells, and disease mutations. Its focus on spatial and temporal characterization makes it distinct from and complementary to other methods such as single-cell RNA sequencing (*7*, *9*) or multiplexed tissue imaging (*65*, *66*). The TypeTracker approach systematically follows all cells in a region of interest, thus allowing direct correlative analysis, and is straightforward to implement, as it requires only confocal microscopy, antibody staining, and the algorithms that we present here. It can also readily be combined with other measurements, such as end point single-molecule fluorescence in situ hybridization and real-time fluorescence microscopy of various cellular processes and signals.

## MATERIALS AND METHODS

### Organoid culture

Murine intestinal organoids carrying both an H2B-mCherry reporter and an Lgr5-GFP reporter were gifts from N. Sachs and J. Beumer, from the group of H.C. in Hubrecht Institute. Organoids were embedded in “domes” of BME (Trevigen) in tissue culture plates. They were further submerged in growth medium consisting of murine recombinant epidermal growth factor (50 ng/ml; Life Technologies), murine recombinant Noggin (100 ng/ml; PeproTech), human recombinant R-spondin 1 (500 ng/ml; Peprotech), *n*-acetylcysteine (1 mM; Sigma-Aldrich), N2 supplement (1×; Life Technologies) and B27 supplement (1×; Life Technologies), GlutaMAX (2 mM; Life Technologies), Hepes (10 mM; Life Technologies), and penicillin-streptomycin (100 U/ml; 100 μg/ml; Life Technologies) in Advanced DMEM/F-12 (Life Technologies). Organoids were kept in incubators at 37°C and with 5% CO_2_. The medium was changed every 2 days. Each week, organoids were passaged by mechanically dissociating crypts using a narrowed glass pipette.

### Organoid sample preparation for imaging

In conventional culture conditions, organoids were embedded in domes of BME droplets and were thus at different heights relative to the plate bottom (fig. S1A). Imaging organoids located far from the plate bottom required long working distance objectives and increased light exposure, leading to excessive phototoxicity. To improve the imaging procedures, we used the four-well chambered cover glass (#1.5 high-performance cover glass) from Cellvis as imaging plates. Organoids were broken into single crypts, seeded in the imaging plates, and put in the refrigerator (~4°C) for ~10 min, allowing them to sink downward to the cover glass. Afterward, they would be incubated at 37°C with 5% CO_2_ for 20 min so that the gel could solidify with organoids settled at the bottom of the wells (fig. S1B). Growth medium was added after the incubation. Organoids were then kept in the incubator for around 2 days until the imaging experiments. For the enrichment of goblet cells, 5 μM IWP2 and 10 μM DAPT were added to the medium.

### Time-lapse imaging with 3D confocal microscope

Time-lapse imaging was performed with a scanning confocal microscope (Leica TCS SP8) with a 40× water immersion objective (numerical aperture, 1.10). Experiments were performed at 37°C and 5% CO_2_. More than 20 organoids with already budded crypts were selected for imaging. Stacks of ~30 *z*-slices with 2-μm step size were taken every 12 min per organoid. Imaging of H2B-mCherry was conducted with an excitation laser of 552 nm at 1% of the laser power, and the emission signals were collected with Leica HyD hybrid detectors whose filter range was set to be 557 to 789 nm.

### Live-cell tracking

Live-cell tracking was conducted by OrganoidTracker, a software developed by our group (*19*). The positions of each nucleus were predicted with a trained neural network, and cells were then automatically linked between frames on the basis of the relative positions and nuclear sizes. The software could report warnings when the linking was less reliable and allowed for manual corrections.

### 3D reconstruction

3D reconstructions of organoids were made with Blender, a free and open-source 3D computer graphics software. Each cell was represented by a 3D sphere and could be colored on the basis of the (inferred) cell types (Fig. 1, B and F).

### Organoid fixation and permeabilization

Organoid samples were fixed with 4% formaldehyde (Sigma-Aldrich) at room temperature. To get rid of the gel but keep the organoids attached to the plate, we optimized the fixation protocol. After adding formaldehyde, we waited for ~10 min and then gently washed the sample with phosphate-buffered saline to remove the gel, which otherwise would hinder the penetration of antibodies and reduce imaging quality. Ten minutes was the optimized waiting time to ensure gel removal, and more than 50% of the imaged organoids attached to the cover glass (fig. S1C). After gel removal, organoid samples were incubated in formaldehyde again for 20 min to complete the fixation procedures. Following fixation, permeabilization was performed by incubating the samples in 0.2% Triton X-100 (Sigma-Aldrich) for 1 hour at room temperature. All the washing procedures were performed gently to avoid removing organoids from the cover glass.

### Staining with antibodies and dyes

Following fixation and permeabilization, organoids were blocked with 5% skim milk in tris-buffered saline at room temperature for 1 hour. Subsequently, organoids were incubated in blocking buffer containing primary antibody (see the “Antibodies and dyes” section) for 2 days at 4°C and then incubated with secondary antibody (see the “Antibodies and dyes” section) at room temperature for 1 hour. These procedures would be repeated for each antibody. Regarding the dyes, organoids were incubated with WGA conjugated to CF488A (5 μg/ml; Biotium) at room temperature for 2 hours and with RedDot1 Far-Red Nuclear stain (1:200; Biotium) or SYTOX Orange Nucleic Acid stain (1:5000; Thermo Fisher Scientific, #S11368) at room temperature for 20 min.

### Antibody stripping

After imaging the results from each round of antibody staining, the primary antibodies were removed by incubation with elution buffer at room temperature for 15 min while shaking (*10*). This was repeated six times with the elution buffer replaced between consecutive cycles. The elution buffer was prepared by adding 0.5 M glycine (Sigma-Aldrich), 5 M urea (Sigma-Aldrich), 5 M guanidinium chloride (Sigma-Aldrich), and 70 mM Tris (2-carboxyethyl) phosphine (TCEP)-HCl (Sigma-Aldrich) to H_2_O, with pH adjusted to 2.5.

### Antibodies and dyes

The primary antibodies used in this study are as follows: rabbit anti-Lyz (1:800; Dako, #A0099), rabbit anti-Olfm4 (1:500; Cell Signaling Technology, #39141), recombinant rabbit anti-aldolase B + aldolase C (1:300; Abcam, #ab75751), mouse anti-human KRT20 (1:500; Dako, #M701929-2), mouse anti–Chr-A (1:50; Santa Cruz Biotechnology, #sc-393941), rat anti–E-cadherin (1:400; Santa Cruz Biotechnology, #sc-59778), CD24 Monoclonal Antibody (1:200; Thermo Fisher Scientific, #17-0242-80), and mouse anti-Ki67 (1:200; BD Biosciences, 550609). The secondary antibodies used in this study are as follows: Goat Anti-Rabbit IgG H&L (Alexa Fluor 405) preadsorbed (1:1000; Abcam, #ab175654), Goat Anti-Rat IgG H&L (Alexa Fluor 555) preadsorbed (1:1000; Abcam, #ab150166), Donkey Anti-Mouse IgG H&L (Alexa Fluor 647) (1:500; Thermo Fisher Scientific, #A31571), and Donkey Anti-Rabbit IgG H&L (Alexa Fluor 405) preadsorbed (1:1000; Abcam, #ab175649). The dyes used in this study are as follows: WGA conjugated to CF488A (5 μg/ml; Biotium), RedDot1 Far-Red Nuclear stain (1:200; Biotium), and SYTOX Orange Nucleic Acid Stain (1:5000; Thermo Fisher Scientific, #S11368). The order of staining, optimized to ensure good staining quality for all cell types, was based on the staining quality and stripping difficulty of each antibody (fig. S1G).

### Mapping end point cell types to lineages

During time-lapse imaging, the Leica software allowed recording of the imaged locations, which could thus be found back after imaging. To achieve this, the mounting stage of the microscope and the orientation of the cover glass should be consistent with the settings during time-lapse imaging. With our optimized protocols for sample preparation and fixation (see the “Organoid fixation and permeabilization” section), we could keep more than 50% of the imaged organoids with limited deformations in the plate after fixation. After staining and relocating the organoids that were imaged in time during growth, mapping all cells (including their type information) to the cells that were tracked could be still challenging because of the constant movement of cells during growth and global rotation and deformation of organoids caused by the fixation and repeated staining and washing. To mitigate these issues, we fixed the organoids within 5 min after the time-lapse imaging and performed every washing step gently to preserve the spatial context of single cells. The linking of cells before and after fixation could be achieved mostly on the basis of the spatial context of each cell. Linking was done in two steps. In the first automated step, we used a minimum-cost flow solver algorithm (*42*), which integrally optimizes the linking for all the tracked cells and was also used for the similar task of tracking cells between frames during organoid growth. In the second step, we manually corrected the automated linking results by visual inspection of the movie and staining images. The fluorescence intensity of H2B-mCherry showed heterogeneity between cells during time-lapse imaging, which could be preserved during fixation. Therefore, the brightness of the nuclear marker could also assist cell linking before and after fixation.

### End point sister type analysis

For cells present at the end point, identified as specific type and with a sister, we checked the possible cell types of the sister pairs and counted the occurrence of each combination. For each cell type, we counted the number of sister pairs where at least one of them was of that type. If both sisters were of that type, the pair would be counted twice. Cells of different types had different abundance, and majority of the sister pairs contained stem cells and/or TA cells. We then normalized the 2D histogram via dividing the occurrence of each combination by the sum of each column, as shown in the bar plot in Fig. 2A. Therefore, the frequency within each column in Fig. 2A would sum up to be 1. The analysis was based on nine different organoids.

### Cell age distribution analysis

For cells present at the end point, the duration between the birth time and the end point of imaging was measured as the cell age. Some cells were present from the beginning until the end. Their ages were then measured by the total length of the imaging experiment duration (~60 hours). The age distribution of each cell type was studied and plotted as a box plot in Fig. 2B, followed by statistical significance tests. Box plot elements represent the following: center line, median; box, quartiles; whiskers, range; and fliers, outliers. The analysis was based on seven different organoids from experiments lasting ~60 hours.

### Analysis within (sub-)trees containing two cell types

All the (sub-)lineage trees with two different cell types were taken into account, unless more than 50% of the cells within the lineage could not be tracked or died. The absolute count of each possible combination of the two cell types was shown in the 2D histogram (fig. S10).

### Cell type backpropagation

The assumption underlying the backpropagation of cell types is that changes in cell types are rare. This assumption could be supported by the found type symmetry between sisters because frequent type changes likely lead to different types in sisters. Starting at the lineage end points, we propagate the measured end point types back in time following this process:

1) Backpropagation along consecutive time points. From one time point to a previous time point, the type is initially assigned as unchanged if no tree branch point (divisions) is traversed (marked “1” in Fig. 2C).

2) Backpropagation of symmetric fate. For cell types identified by end point staining, we observed that sisters almost always assumed the same fate (Fig. 2A), suggesting that this fate was already set in the mother cell. Generalizing this observation, we assumed that if both sisters have the same (inferred) cell type, the inferred cell type of the mother cell is the same (marked “2” in Fig. 2C). Regarding cells with a dead sister, the mother is inferred the same type as the living daughter.

3) Backpropagation of asymmetric fate. The above backpropagation rule does not apply if two daughters have different (inferred) cell types. Therefore, we introduced two additional backpropagation rules. First, if at least one daughter’s (inferred) cell type was stem cell, then the inferred cell type of the mother was also stem cell (marked “3” in Fig. 2C). Second, if the (inferred) cell type of one daughter was TA and the other daughter was not stem cell, then the inferred cell type of the mother was TA (Fig. 2D). These two rules were based on the capability of stem cells to generate all cell types and the transient property of TA cells between stem cells and differentiated cells.

4) Forward propagation of cell type changes. If a mother and a daughter cell had different (inferred) cell types, we interpreted this as a change in cell type that occurred during the lifetime of the daughter cell [marked with triangle in Fig. 2 (C and D)].

These simple rules were sufficient to propagate backward the lineage trees that we have encountered, unless the tree appeared very “broken” where majority of the cells could not be tracked or died. All the lineage trees after backpropagation were shown in fig. S7.

### Imaging of an Lgr5 reporter

To test our backpropagation method, we performed time-lapse imaging, end point staining, and live-cell tracking in an organoid line with both Lgr5-GFP, a well-known stem cell marker, and an H2B-mCherry reporter. To limit phototoxicity caused by GFP imaging, the time-lapse imaging lasted for around 24 hours, followed by end point staining. Quantification of the membrane-bound Lgr5-GFP fluorescence signals was conducted by determining the average fluorescence intensity within a 2D circle centered around the membrane area with a diameter of 6 μm (fig. S8A). The Olfm4 staining was measured with the same method.

### Imaging of Paneth cells and EECs

To image Paneth cells, CD24 antibody was added to the medium of H2B-mCherry organoids 1 hour before imaging. Time-lapse imaging was performed in both H2B-mCherry and CD24 (647 nm) channels for more than 24 hours. To image EECs, a mouse organoid line carrying a Neurogenin 3–tdTomato reporter was used (*22*).

### Measuring locations of cells along the crypt-villus axis

At each time point, the crypt-villus axis was manually annotated in the *xy* plane at the *z* position corresponding to the center of the crypt because tracked crypts grew perpendicularly to the objective. Three to six points were marked along the axis, through which a spline curve was interpolated as the axis. For each tracked cell *i*, we determined its position along the spline by finding the value of *r_i_* that minimized the distance *d* between the cell position and the axis (fig. S12A). The bottom-most cell of the crypt, i.e., that with the lowest value of *r_i_*, was defined as position zero. On the basis of the shape and curvature of the epithelium, the location of crypt neck (where there was a sharp transition from crypt to villus) was estimated and annotated manually, as an indication of the length of the crypt. Because different crypts were of various length, we did a normalization of the locations based on the crypt neck location. For each cell’s measured distance in micrometers within a certain frame, we divided it by the distance from the crypt neck within the same frame to the crypt bottom. Therefore, the length from the crypt neck to the bottom would remain one for each time point and each crypt. With this measurement, both the locations of different (inferred) cell types and the type transitions were mapped along the crypt-villus axis (Fig. 3, A to E).

### Measurement of migration speed along the axis

To estimate how fast a cell migrated along the axis, we searched for the locations of cells along the crypt-villus axis when they first showed up during tracking and the locations of cells when they were last present. The migration speed could be estimated by dividing the distance that the cell had migrated by the duration during which the cell was present (Fig. 3F).

### Search for neighbors for each cell

Cells could have varying numbers of neighbors because of the disorder in the epithelium. Distances between nuclei could vary between cell types and location (spread apart in the villus-like region and closely packed in the crypts). To obtain robust neighbor pairs, we functionally defined neighbors as pairs of nuclei without another nucleus in between (fig. S12D). This condition was tested by a “neighbor score,” the ratio of the sum of the distances of the two cells of interest (A and B) to a third cell (S) and the distance between the two cells (A and B), namely, $\frac{d_{\mathrm{AS}}+d_{\mathrm{BS}}}{d_{\mathrm{AB}}}$. If the third cell S positioned perfectly in between the pair of interest A and B, then the neighbor score would appear as the minimal value of 1, and A and B would not be identified as neighbors. If A and B were not separated by S, then the three nuclei would form a triangle with high neighbor score between A and B. For each cell, we calculated the neighbor score for the twenty closest neighbors (in Euclidean distance) at every time point. If the neighbor score were higher than $\sqrt{2}$, we would consider them neighbors. This cutoff corresponded to diagonal neighbors in the case of a perfect square lattice. Using this cutoff, we found most cells with five or six neighbors, exactly as expected for the basal side of a curved epithelium (*67*).

### Measurement of separation rate

Separation rates were determined by following pairs of neighbors over time. For a newly born cell, its neighbors were searched and selected with the method introduced above. The selection was conducted 1 hour after division so that the nuclei would have returned to the basal side of the epithelium. If the selected neighbors divided, then we would continue tracking one of the daughters (selected randomly) so that the following of the neighbor pairs would not be cut short by division. The separation rates were measured after following the neighbor pairs for 2 and 10 hours, by calculating the fraction of the pairs staying as direct neighbors within the total of pairs that were followed. Regarding the rearrangement rates of sisters, we followed the sister pairs that shared the same (inferred) cell type. Separation rates of sister pairs were also measured after 2 and 10 hours by calculating the fraction of sisters staying as direct neighbors within the total number of sister pairs being followed (Fig. 3, H and I).

## References

[1] T. Sato, R. G. Vries, H. J. Snippert, M. van de Wetering, N. Barker, D. E. Stange, J. H. van Es, A. Abo, P. Kujala, P. J. Peters, H. Clevers, Single Lgr5 stem cells build crypt-villus structures in vitro without a mesenchymal niche. Nature 459, 262–265 (2009). 19329995 10.1038/nature07935

[2] M. A. Lancaster, M. Renner, C.-A. Martin, D. Wenzel, L. S. Bicknell, M. E. Hurles, T. Homfray, J. M. Penninger, A. P. Jackson, J. A. Knoblich, Cerebral organoids model human brain development and microcephaly. Nature 501, 373–379 (2013). 23995685 10.1038/nature12517 PMC3817409

[3] A. J. Miller, B. R. Dye, D. Ferrer-Torres, D. R. Hill, A. W. Overeem, L. D. Shea, J. R. Spence, Generation of lung organoids from human pluripotent stem cells in vitro. Nat. Protoc. 14, 518–540 (2019). 30664680 10.1038/s41596-018-0104-8 PMC6531049

[4] H. Clevers, Modeling development and disease with organoids. Cell 165, 1586–1597 (2016). 27315476 10.1016/j.cell.2016.05.082

[5] H. Hu, H. Gehart, B. Artegiani, C. LÖpez-Iglesias, F. Dekkers, O. Basak, J. van Es, S. M. C. de Sousa Lopes, H. Begthel, J. Korving, M. van den Born, C. Zou, C. Quirk, L. Chiriboga, C. M. Rice, S. Ma, A. Rios, P. J. Peters, Y. P. de Jong, H. Clevers, Long-term expansion of functional mouse and human hepatocytes as 3D organoids. Cell 175, 1591–1606.e19 (2018). 30500538 10.1016/j.cell.2018.11.013

[6] I. Lukonin, D. Serra, L. Challet Meylan, K. Volkmann, J. Baaten, R. Zhao, S. Meeusen, K. Colman, F. Maurer, M. B. Stadler, J. Jenkins, P. Liberali, Phenotypic landscape of intestinal organoid regeneration. Nature 586, 275–280 (2020). 33029001 10.1038/s41586-020-2776-9 PMC7116869

[7] D. Grün, A. Lyubimova, L. Kester, K. Wiebrands, O. Basak, N. Sasaki, H. Clevers, A. van Oudenaarden, Single-cell messenger RNA sequencing reveals rare intestinal cell types. Nature 525, 251–255 (2015). 26287467 10.1038/nature14966

[8] J. G. Camp, F. Badsha, M. Florio, S. Kanton, T. Gerber, M. Wilsch-Bräuninger, E. Lewitus, A. Sykes, W. Hevers, M. Lancaster, A. Knoblich Juergen, R. Lachmann, S. Pääbo, W. B. Huttner, B. Treutlein, Human cerebral organoids recapitulate gene expression programs of fetal neocortex development. Proc. Natl. Acad. Sci. U.S.A. 112, 15672–15677 (2015). 26644564 10.1073/pnas.1520760112 PMC4697386

[9] A. L. Haber, M. Biton, N. Rogel, R. H. Herbst, K. Shekhar, C. Smillie, G. Burgin, T. M. Delorey, M. R. Howitt, Y. Katz, I. Tirosh, S. Beyaz, D. Dionne, M. Zhang, R. Raychowdhury, W. S. Garrett, O. Rozenblatt-Rosen, H. N. Shi, O. Yilmaz, R. J. Xavier, A. Regev, A single-cell survey of the small intestinal epithelium. Nature 551, 333–339 (2017). 29144463 10.1038/nature24489 PMC6022292

[10] D. Serra, U. Mayr, A. Boni, I. Lukonin, M. Rempfler, L. Challet Meylan, M. B. Stadler, P. Strnad, P. Papasaikas, D. Vischi, A. Waldt, G. Roma, P. Liberali, Self-organization and symmetry breaking in intestinal organoid development. Nature 569, 66–72 (2019). 31019299 10.1038/s41586-019-1146-y PMC6544541

[11] K. Kretzschmar, F. M. Watt, Lineage tracing. Cell 148, 33–45 (2012). 22265400 10.1016/j.cell.2012.01.002

[12] H. J. Snippert, L. G. van der Flier, T. Sato, J. H. van Es, M. van den Born, C. Kroon-Veenboer, N. Barker, A. M. Klein, J. van Rheenen, B. D. Simons, H. Clevers, Intestinal crypt homeostasis results from neutral competition between symmetrically dividing Lgr5 stem cells. Cell 143, 134–144 (2010). 20887898 10.1016/j.cell.2010.09.016

[13] B. Tóth, S. Ben-Moshe, A. Gavish, N. Barkai, S. Itzkovitz, Early commitment and robust differentiation in colonic crypts. Mol. Syst. Biol. 13, 902 (2017). 28049136 10.15252/msb.20167283 PMC5293156

[14] L. E. Sanman, I. W. Chen, J. M. Bieber, V. Steri, C. Trentesaux, B. Hann, O. D. Klein, L. F. Wu, S. J. Altschuler, Transit-amplifying cells coordinate changes in intestinal epithelial cell-type composition. Dev. Cell 56, 356–365.e9 (2021). 33484640 10.1016/j.devcel.2020.12.020 PMC7917018

[15] A. C. Rios, H. Clevers, Imaging organoids: A bright future ahead. Nat. Methods 15, 24–26 (2018). 29298292 10.1038/nmeth.4537

[16] Y. Yue, W. Zong, X. Li, J. Li, Y. Zhang, R. Wu, Y. Liu, J. Cui, Q. Wang, Y. Bian, X. Yu, Y. Liu, G. Tan, Y. Zhang, G. Zhao, B. Zhou, L. Chen, W. Xiao, H. Cheng, A. He, Long-term, in toto live imaging of cardiomyocyte behaviour during mouse ventricle chamber formation at single-cell resolution. Nat. Cell Biol. 22, 332–340 (2020). 32123336 10.1038/s41556-020-0475-2

[17] K. McDole, L. Guignard, F. Amat, A. Berger, G. Malandain, L. A. Royer, S. C. Turaga, K. Branson, P. J. Keller, In toto imaging and reconstruction of post-implantation mouse development at the single-cell level. Cell 175, 859–876.e33 (2018). 30318151 10.1016/j.cell.2018.09.031

[18] N. P. Tallapragada, H. M. Cambra, T. Wald, S. Keough Jalbert, D. M. Abraham, O. D. Klein, A. M. Klein, Inflation-collapse dynamics drive patterning and morphogenesis in intestinal organoids. Cell Stem Cell 28, 1516–1532.e14 (2021). 33915079 10.1016/j.stem.2021.04.002 PMC8419000

[19] R. N. U. Kok, L. Hebert, G. Huelsz-Prince, Y. J. Goos, X. Zheng, K. Bozek, G. J. Stephens, S. J. Tans, J. S. van Zon, OrganoidTracker: Efficient cell tracking using machine learning and manual error correction. PLOS ONE 15, e0240802 (2020). 33091031 10.1371/journal.pone.0240802 PMC7580893

[20] Z. He, A. Maynard, A. Jain, T. Gerber, R. Petri, H.-C. Lin, M. Santel, K. Ly, J.-S. Dupré, L. Sidow, F. Sanchis Calleja, S. M. J. Jansen, S. Riesenberg, J. G. Camp, B. Treutlein, Lineage recording in human cerebral organoids. Nat. Methods 19, 90–99 (2022). 34969984 10.1038/s41592-021-01344-8 PMC8748197

[21] K. Sugawara, Ç. Çevrim, M. Averof, Tracking cell lineages in 3D by incremental deep learning. eLife 11, e69380 (2022). 34989675 10.7554/eLife.69380 PMC8741210

[22] H. Gehart, J. H. van Es, K. Hamer, J. Beumer, K. Kretzschmar, J. F. Dekkers, A. Rios, H. Clevers, Identification of enteroendocrine regulators by real-time single-cell differentiation mapping. Cell 176, 1158–1173.e16 (2019). 30712869 10.1016/j.cell.2018.12.029

[23] B. Artegiani, D. Hendriks, J. Beumer, R. Kok, X. Zheng, I. Joore, S. C. de Sousa Lopes, J. van Zon, S. Tans, H. Clevers, Fast and efficient generation of knock-in human organoids using homology-independent CRISPR–Cas9 precision genome editing. Nat. Cell Biol. 22, 321–331 (2020). 32123335 10.1038/s41556-020-0472-5

[24] K. F. Sonnen, V. M. Lauschke, J. Uraji, H. J. Falk, Y. Petersen, M. C. Funk, M. Beaupeux, P. François, C. A. Merten, A. Aulehla, Modulation of phase shift between Wnt and Notch signaling oscillations controls mesoderm segmentation. Cell 172, 1079–1090.e12 (2018). 29474908 10.1016/j.cell.2018.01.026 PMC5847172

[25] M. A. Betjes, X. Zheng, R. N. U. Kok, J. S. van Zon, S. J. Tans, Cell tracking for organoids: Lessons from developmental biology. Front. Cell Dev. Biol. 9, 675013 (2021). 34150770 10.3389/fcell.2021.675013 PMC8209328

[26] J. Beumer, H. Clevers, Cell fate specification and differentiation in the adult mammalian intestine. Nat. Rev. Mol. Cell Biol. 22, 39–53 (2021). 32958874 10.1038/s41580-020-0278-0

[27] L. G. van der Flier, H. Clevers, Stem cells, self-renewal, and differentiation in the intestinal epithelium. Annu. Rev. Physiol. 71, 241–260 (2009). 18808327 10.1146/annurev.physiol.010908.163145

[28] C. Capdevila, M. Trifas, J. Miller, T. Anderson, P. A. Sims, K. S. Yan, Cellular origins and lineage relationships of the intestinal epithelium. Am. J. Physiol. Gastrointerest. Liver Physiol. 321, G413–G425 (2021). 10.1152/ajpgi.00188.2021 PMC8560372 34431400

[29] H. Cheng, C. P. Leblond, Origin, differentiation and renewal of the four main epithelial cell types in the mouse small intestine V. Unitarian theory of the origin of the four epithelial cell types. Am. J. Anat. 141, 537–561 (1974). 4440635 10.1002/aja.1001410407

[30] T. Fevr, S. Robine, D. Louvard, J. Huelsken, Wnt/β-catenin is essential for intestinal homeostasis and maintenance of intestinal stem cells. Mol. Cell. Biol. 27, 7551–7559 (2007). 17785439 10.1128/MCB.01034-07 PMC2169070

[31] M. Spit, B.-K. Koo, M. M. Maurice, Tales from the crypt: Intestinal niche signals in tissue renewal, plasticity and cancer. Open Biol. 8, 180120 (2018). 30209039 10.1098/rsob.180120 PMC6170508

[32] S. Fre, M. Huyghe, P. Mourikis, S. Robine, D. Louvard, S. Artavanis-Tsakonas, Notch signals control the fate of immature progenitor cells in the intestine. Nature 435, 964–968 (2005). 15959516 10.1038/nature03589

[33] S. J. A. Buczacki, H. I. Zecchini, A. M. Nicholson, R. Russell, L. Vermeulen, R. Kemp, D. J. Winton, Intestinal label-retaining cells are secretory precursors expressing Lgr5. Nature 495, 65–69 (2013). 23446353 10.1038/nature11965

[34] O. Basak, M. van de Born, J. Korving, J. Beumer, S. van der Elst, J. H. van Es, H. Clevers, Mapping early fate determination in Lgr5+ crypt stem cells using a novel Ki67-RFP allele. EMBO J. 33, 2057–2068 (2014). 25092767 10.15252/embj.201488017 PMC4195772

[35] D. Stamataki, M. Holder, C. Hodgetts, R. Jeffery, E. Nye, B. Spencer-Dene, D. J. Winton, J. Lewis, Delta1 expression, cell cycle exit, and commitment to a specific secretory fate coincide within a few hours in the mouse intestinal stem cell system. PLOS ONE 6, e24484 (2011). 21915337 10.1371/journal.pone.0024484 PMC3168508

[36] J. Heuberger, F. Kosel, J. Qi, K. S. Grossmann, K. Rajewsky, W. Birchmeier, Shp2/MAPK signaling controls goblet/paneth cell fate decisions in the intestine. Proc. Natl. Acad. Sci. U.S.A. 111, 3472–3477 (2014). 24550486 10.1073/pnas.1309342111 PMC3948231

[37] N. F. Shroyer, D. Wallis, K. J. T. Venken, H. J. Bellen, H. Y. Zoghbi, Gfi1 functions downstream of Math1 to control intestinal secretory cell subtype allocation and differentiation. Genes Dev. 19, 2412–2417 (2005). 16230531 10.1101/gad.1353905 PMC1257395

[38] J. H. van Es, T. Sato, M. van de Wetering, A. Lyubimova, A. N. Yee Nee, A. Gregorieff, N. Sasaki, L. Zeinstra, M. van den Born, J. Korving, A. C. M. Martens, N. Barker, A. van Oudenaarden, H. Clevers, Dll1+ secretory progenitor cells revert to stem cells upon crypt damage. Nat. Cell Biol. 14, 1099–1104 (2012). 23000963 10.1038/ncb2581 PMC3789123

[39] S. E. Schonhoff, M. Giel-Moloney, A. B. Leiter, Neurogenin 3-expressing progenitor cells in the gastrointestinal tract differentiate into both endocrine and non-endocrine cell types. Dev. Biol. 270, 443–454 (2004). 15183725 10.1016/j.ydbio.2004.03.013

[40] H. Clevers, The intestinal crypt, a prototype stem cell compartment. Cell 154, 274–284 (2013). 23870119 10.1016/j.cell.2013.07.004

[41] V. Bonis, C. Rossell, H. Gehart, The intestinal epithelium—Fluid fate and rigid structure from crypt bottom to villus tip. Front. Cell Dev. Biol. 9, 661931 (2021). 34095127 10.3389/fcell.2021.661931 PMC8172987

[42] C. Haubold, J. Aleš, S. Wolf, F. A. Hamprecht, in *Computer Vision—ECCV 2016*, B. Leibe, J. Matas, N. Sebe, M. Welling, Eds. (Springer International Publishing, 2016), pp. 566–582.

[43] L. G. van der Flier, A. Haegebarth, D. E. Stange, M. van de Wetering, H. Clevers, OLFM4 is a robust marker for stem cells in human intestine and marks a subset of colorectal cancer cells. Gastroenterology 137, 15–17 (2009). 19450592 10.1053/j.gastro.2009.05.035

[44] O. Louthan, Chromogranin a in physiology and oncology. Folia Biol. (Praha) 57, 173–181 (2011). 22123459 10.14712/fb2011057050173

[45] C. W. M. Chan, N. A. Wong, Y. Liu, D. Bicknell, H. Turley, L. Hollins, C. J. Miller, J. L. Wilding, W. F. Bodmer, Gastrointestinal differentiation marker Cytokeratin 20 is regulated by homeobox gene CDX1. Proc. Natl. Acad. Sci. U.S.A. 106, 1936–1941 (2009). 19188603 10.1073/pnas.0812904106 PMC2644142

[46] S. Bel, M. Pendse, Y. Wang, Y. Li, K. A. Ruhn, B. Hassell, T. Leal, S. E. Winter, R. J. Xavier, L. V. Hooper, Paneth cells secrete lysozyme via secretory autophagy during bacterial infection of the intestine. Science 357, 1047–1052 (2017). 28751470 10.1126/science.aal4677 PMC5702267

[47] M. Kim, C. Fevre, M. Lavina, O. Disson, M. Lecuit, Live imaging reveals listeria hijacking of E-cadherin recycling as it crosses the intestinal barrier. Curr. Biol. 31, 1037–1047.e4 (2021). 33333010 10.1016/j.cub.2020.11.041

[48] T. Sato, J. H. van Es, H. J. Snippert, D. E. Stange, R. G. Vries, M. van den Born, N. Barker, N. F. Shroyer, M. van de Wetering, H. Clevers, Paneth cells constitute the niche for Lgr5 stem cells in intestinal crypts. Nature 469, 415–418 (2011). 21113151 10.1038/nature09637 PMC3547360

[49] X. Yin, H. F. Farin, J. H. van Es, H. Clevers, R. Langer, J. M. Karp, Niche-independent high-purity cultures of Lgr5+ intestinal stem cells and their progeny. Nat. Methods 11, 106–112 (2014). 24292484 10.1038/nmeth.2737 PMC3951815

[50] N. Barker, Adult intestinal stem cells: Critical drivers of epithelial homeostasis and regeneration. Nat. Rev. Mol. Cell Biol. 15, 19–33 (2014). 24326621 10.1038/nrm3721

[51] N. Barker, J. H. van Es, J. Kuipers, P. Kujala, M. van den Born, M. Cozijnsen, A. Haegebarth, J. Korving, H. Begthel, P. J. Peters, H. Clevers, Identification of stem cells in small intestine and colon by marker gene Lgr5. Nature 449, 1003–1007 (2007). 17934449 10.1038/nature06196

[52] Q. Yang, N. A. Bermingham, M. J. Finegold, H. Y. Zoghbi, Requirement of Math1 for secretory cell lineage commitment in the mouse intestine. Science 294, 2155–2158 (2001). 11739954 10.1126/science.1065718

[53] K. L. McKinley, N. Stuurman, L. A. Royer, C. Schartner, D. Castillo-Azofeifa, M. Delling, O. D. Klein, R. D. Vale, Cellular aspect ratio and cell division mechanics underlie the patterning of cell progeny in diverse mammalian epithelia. eLife 7, e36739 (2018). 29897330 10.7554/eLife.36739 PMC6023609

[54] A. Böttcher, M. Büttner, S. Tritschler, M. Sterr, A. Aliluev, L. Oppenländer, I. Burtscher, S. Sass, M. Irmler, J. Beckers, C. Ziegenhain, W. Enard, A. C. Schamberger, F. M. Verhamme, O. Eickelberg, F. J. Theis, H. Lickert, Non-canonical Wnt/PCP signalling regulates intestinal stem cell lineage priming towards enteroendocrine and Paneth cell fates. Nat. Cell Biol. 23, 23–31 (2021). 33398177 10.1038/s41556-020-00617-2

[55] E. Batlle, J. T. Henderson, H. Beghtel, M. M. W. van den Born, E. Sancho, G. Huls, J. Meeldijk, J. Robertson, M. van de Wetering, T. Pawson, H. Clevers, β-Catenin and TCF mediate cell positioning in the intestinal epithelium by controlling the expression of EphB/EphrinB. Cell 111, 251–263 (2002). 12408869 10.1016/s0092-8674(02)01015-2

[56] M. Azkanaz, B. Corominas-Murtra, S. I. J. Ellenbroek, L. Bruens, A. T. Webb, D. Laskaris, K. C. Oost, S. J. A. Lafirenze, K. Annusver, H. A. Messal, S. Iqbal, D. J. Flanagan, D. J. Huels, F. Rojas-Rodríguez, M. Vizoso, M. Kasper, O. J. Sansom, H. J. Snippert, P. Liberali, B. D. Simons, P. Katajisto, E. Hannezo, J. van Rheenen, Retrograde movements determine effective stem cell numbers in the intestine. Nature 607, 548–554 (2022). 35831497 10.1038/s41586-022-04962-0 PMC7614894

[57] M. L. Zhao, A. Rabiee, K. M. Kovary, Z. Bahrami-Nejad, B. Taylor, M. N. Teruel, Molecular competition in G1 controls when cells simultaneously commit to terminally differentiate and exit the cell cycle. Cell Rep. 31, 107769 (2020). 32553172 10.1016/j.celrep.2020.107769 PMC8198760

[58] R. Sancho, C. A. Cremona, A. Behrens, Stem cell and progenitor fate in the mammalian intestine: Notch and lateral inhibition in homeostasis and disease. EMBO Rep. 16, 571–581 (2015). 25855643 10.15252/embr.201540188 PMC4428041

[59] H. F. Farin, J. H. Van Es, H. Clevers, Redundant sources of Wnt regulate intestinal stem cells and promote formation of Paneth cells. Gastroenterology 143, 1518–1529.e7 (2012). 22922422 10.1053/j.gastro.2012.08.031

[60] Z. Kabiri, G. Greicius, B. Madan, S. Biechele, Z. Zhong, H. Zaribafzadeh, J. A. Edison, Y. Wu, R. Bunte, B. O. Williams, J. Rossant, D. M. Virshup, Stroma provides an intestinal stem cell niche in the absence of epithelial Wnts. Development 141, 2206–2215 (2014). 24821987 10.1242/dev.104976

[61] T.-H. Kim, S. Escudero, R. A. Shivdasani, Intact function of Lgr5 receptor-expressing intestinal stem cells in the absence of Paneth cells. Proc. Natl. Acad. Sci. U.S.A. 109, 3932–3937 (2012). 22355124 10.1073/pnas.1113890109 PMC3309789

[62] J. H. van Es, K. Wiebrands, C. López-Iglesias, M. van de Wetering, L. Zeinstra, M. van den Born, J. Korving, N. Sasaki, P. J. Peters, A. van Oudenaarden, H. Clevers, Enteroendocrine and tuft cells support Lgr5 stem cells on Paneth cell depletion. Proc. Natl. Acad. Sci. U.S.A. 116, 26599–26605 (2019). 31843916 10.1073/pnas.1801888117 PMC6936398

[63] A. Durand, B. Donahue, G. Peignon, F. Letourneur, N. Cagnard, C. Slomianny, C. Perret, N. F. Shroyer, B. Romagnolo, Functional intestinal stem cells after Paneth cell ablation induced by the loss of transcription factor Math1 (Atoh1). Proc. Natl. Acad. Sci. U.S.A. 109, 8965–8970 (2012). 22586121 10.1073/pnas.1201652109 PMC3384132

[64] R. G. H. Lindeboom, L. van Voorthuijsen, K. C. Oost, M. J. Rodríguez-Colman, M. V. Luna-Velez, C. Furlan, F. Baraille, P. W. T. C. Jansen, A. Ribeiro, B. M. T. Burgering, H. J. Snippert, M. Vermeulen, Integrative multi-omics analysis of intestinal organoid differentiation. Mol. Syst. Biol. 14, e8227 (2018). 29945941 10.15252/msb.20188227 PMC6018986

[65] D. Schapiro, H. W. Jackson, S. Raghuraman, J. R. Fischer, V. R. T. Zanotelli, D. Schulz, C. Giesen, R. Catena, Z. Varga, B. Bodenmiller, histoCAT: Analysis of cell phenotypes and interactions in multiplex image cytometry data. Nat. Methods 14, 873–876 (2017). 28783155 10.1038/nmeth.4391 PMC5617107

[66] C. Giesen, H. A. O. Wang, D. Schapiro, N. Zivanovic, A. Jacobs, B. Hattendorf, P. J. Schüffler, D. Grolimund, J. M. Buhmann, S. Brandt, Z. Varga, P. J. Wild, D. Günther, B. Bodenmiller, Highly multiplexed imaging of tumor tissues with subcellular resolution by mass cytometry. Nat. Methods 11, 417–422 (2014). 24584193 10.1038/nmeth.2869

[67] H. F. Gómez, M. S. Dumond, L. Hodel, R. Vetter, D. Iber, 3D cell neighbour dynamics in growing pseudostratified epithelia. eLife 10, e68135 (2021). 34609280 10.7554/eLife.68135 PMC8570695

