# Supplementary material for: Organoid cell fate dynamics in space and time
Source: Sci Adv. 2023 Aug 18;9(33):eadd6480. doi: 10.1126/sciadv.add6480 (PMC10438469; doi:10.1126/sciadv.add6480)
Supplement: Supplementary file 1 — Figs. S1 to S13 [file sciadv.add6480_sm.pdf]

Science Advances
AAAS

Supplementary Materials for

Organoid cell fate dynamics in space and time

Xuan Zheng *et al.*

Corresponding author: Sander J. Tans, s.tans@amolf.nl; Jeroen S. van Zon, j.v.zon@amolf.nl

Sci. Adv. **9**, eadd6480 (2023)
DOI: 10.1126/sciadv.add6480

This PDF file includes:

Figs. S1 to S13

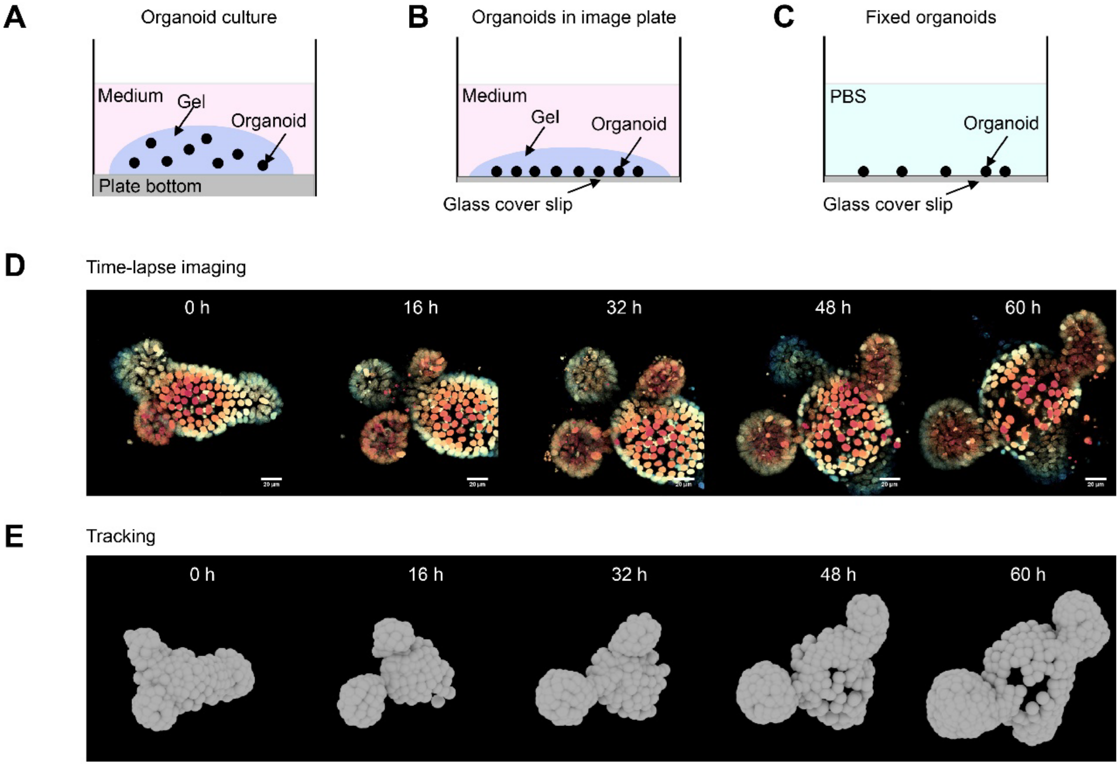

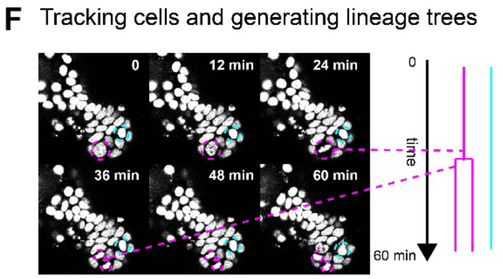

G The optimized staining order

antibody binds to	round	excitation	emission	removal difficulty	staining signal
Olfm4	1	405nm	421nm	Easy	Dim
CgA	1	647nm	675nm	Easy	Good
WGA*	1	488nm	518nm	Easy	Good
Aldoβ	2	405nm	421nm	Easy	Good
KRT20	2	647nm	675nm	Difficult	Good
Lyz	3	405nm	421nm	Difficult	Good

Figure S1. TypeTracker applied to mouse intestinal organoids. A, For conventional organoid culture, organoids were scattered in ‘domes’ of BME gel and located at various heights. **B,** For time-lapse imaging, organoids were seeded in a thin layer of BME gel in chambered cover glass slides. Immediately after seeding, samples were put in fridges for 10 minutes so that organoids all sank towards the cover glass. **C,** With our optimized protocol, more than 50 % of the imaged organoids would remain at their imaged locations after fixation. **D,** Time-lapse imaging of an organoid carrying the H2B-mCherry reporter with 3D confocal for 60 hours. Scale bar, 20 μm. Color encodes different z-planes. **E,** Live-cell tracking of the organoid. In these 3D reconstructions, each cell was represented by a sphere centered at the estimated nuclear center. Cells were not tracked if they were located far away from the objective and would move away from the region of interest. **F,** Tracking cell divisions and generating lineage trees. Cells were tracked between consecutive frames based on their locations, such as the cell highlighted by the cyan circle. Cell division events could be identified by the morphology changes of the nuclei (highlighted with the magenta circle) and recorded with the branching of lineage trees shown in magenta. **G,** The order of antibodies and dyes to use in different rounds was optimized based on each antibody's staining quality and stripping difficulty.

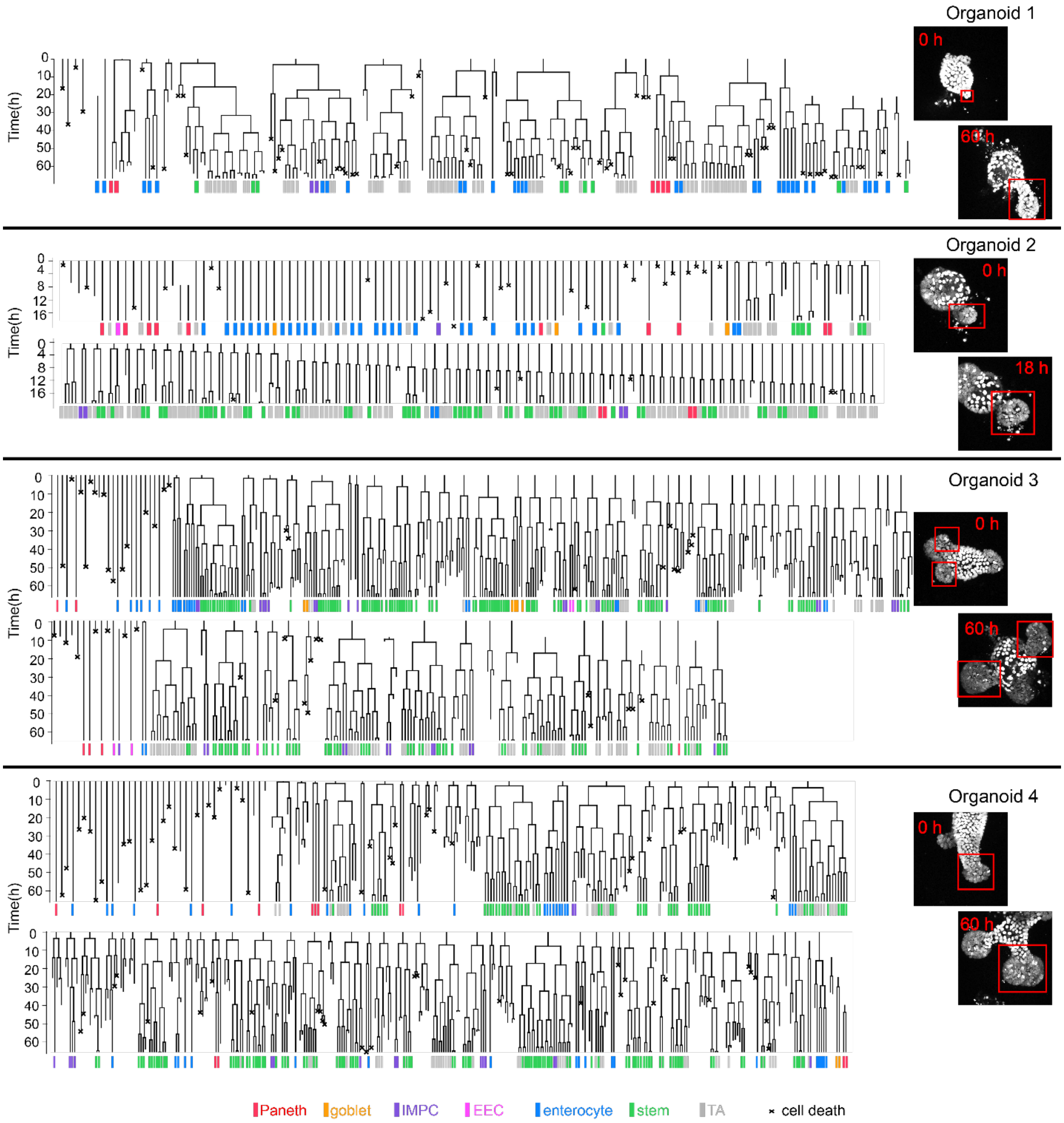

Figure S2. Gallery of lineage trees, generated from live-cell tracking, with cell types mapped at the endpoint. These trees are from four different organoids, of which the max projections of H2B-mCherry signals at the beginning and the end of time-lapse imaging are shown. The regions of tracked cells are marked by red boxes.

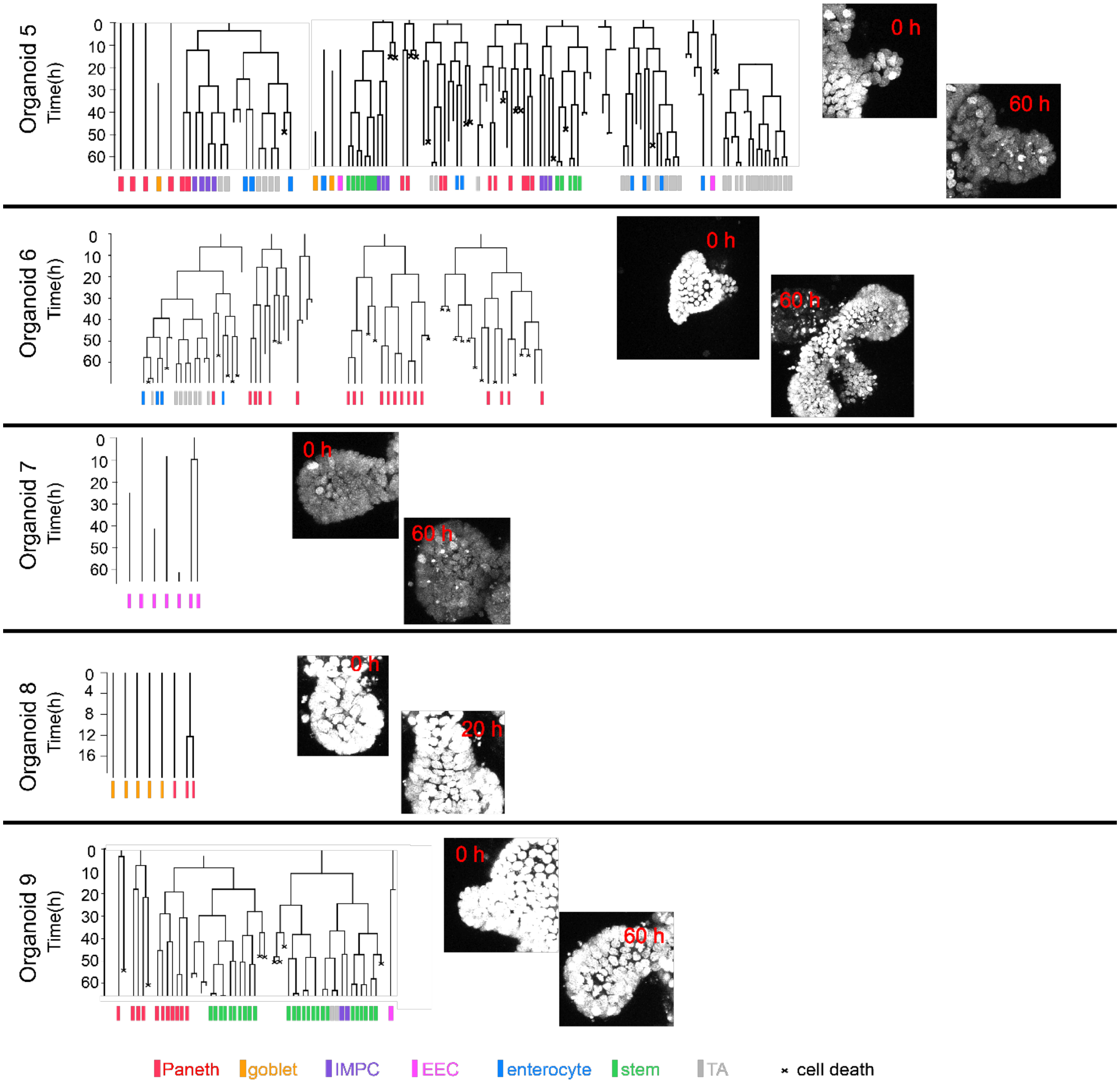

Figure S3. Gallery of lineage trees from organoids where certain cell types were tracked. The max projections of H2B-mCherry signals at the beginning and the end of time-lapse imaging are shown.

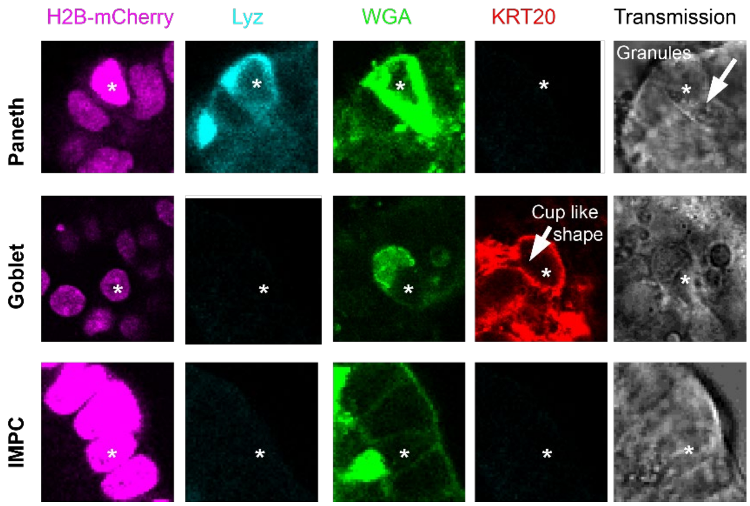

Figure S4. The identification of mature Paneth cells and goblet cells. Paneth cells (indicated with * in row 1) often showed extremely bright H2B-mCherry fluorescence signals compared with the neighbor cells, bright Lysozyme (Lyz) fluorescence signals at the basal side of the cell, bright Wheat Germ Agglutinin (WGA) staining and granules in the transmission channel. Goblet cells (indicated with * in row 2) often stained positive of Cytokeratin 20 (KRT20) and WGA, with a cup-like shape. A group of cells that stained positive of WGA but negative of KRT20 or Lyz were called Immature Mucus producing cells (IMPCs, indicated with * in row 3). These cells could be early Paneth cells or goblet cells considering the mucus secretion functions that they had.

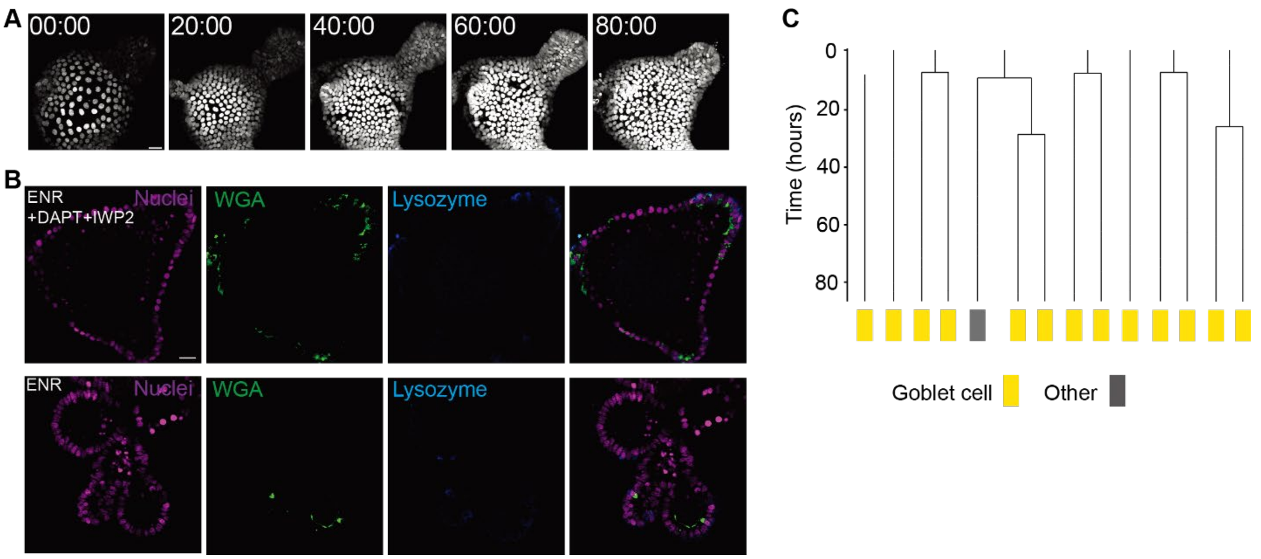

Figure S5. Sister symmetry for goblet cells in enriching conditions. A, Time-lapse imaging of organoids in culture media supplemented with IWP2 and DAPT for 80 hours. **B,** Staining results. **C,** TypeTracker lineages of cells identified as goblet cells at the endpoint by staining. The data are consistent with sister symmetry for secretory types, and early commitment in the mother cell.

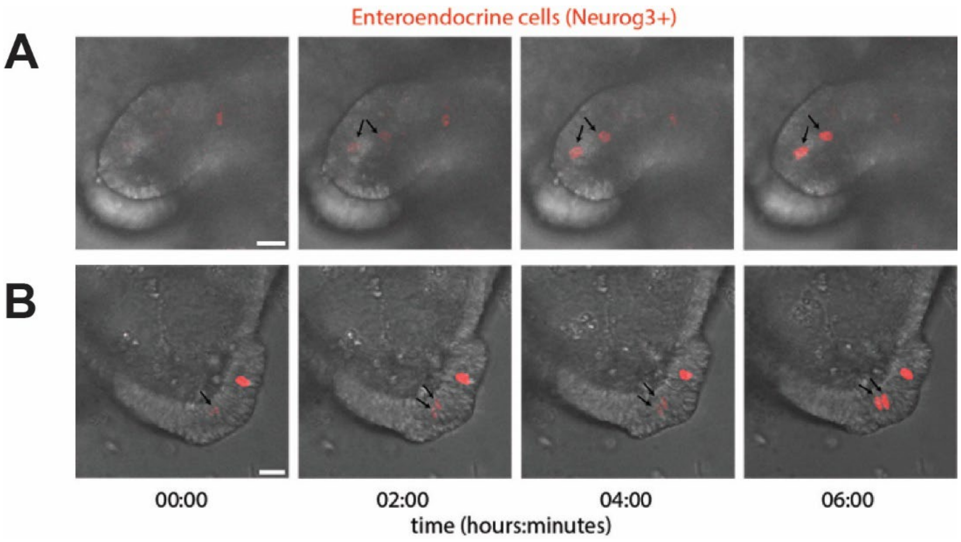

Figure S6. Sister symmetry for enteroendocrine cells visualized with a Neurogenin 3 reporter. Time-lapse imaging of organoids carrying a Neurogenin 3 reporter. **A** and **B** show two examples where cells (marked by arrows) located close to each other started to express Neurogenin 3 signals at a similar time. The data are consistent with sister symmetry for secretory types, and early commitment in the mother cell.

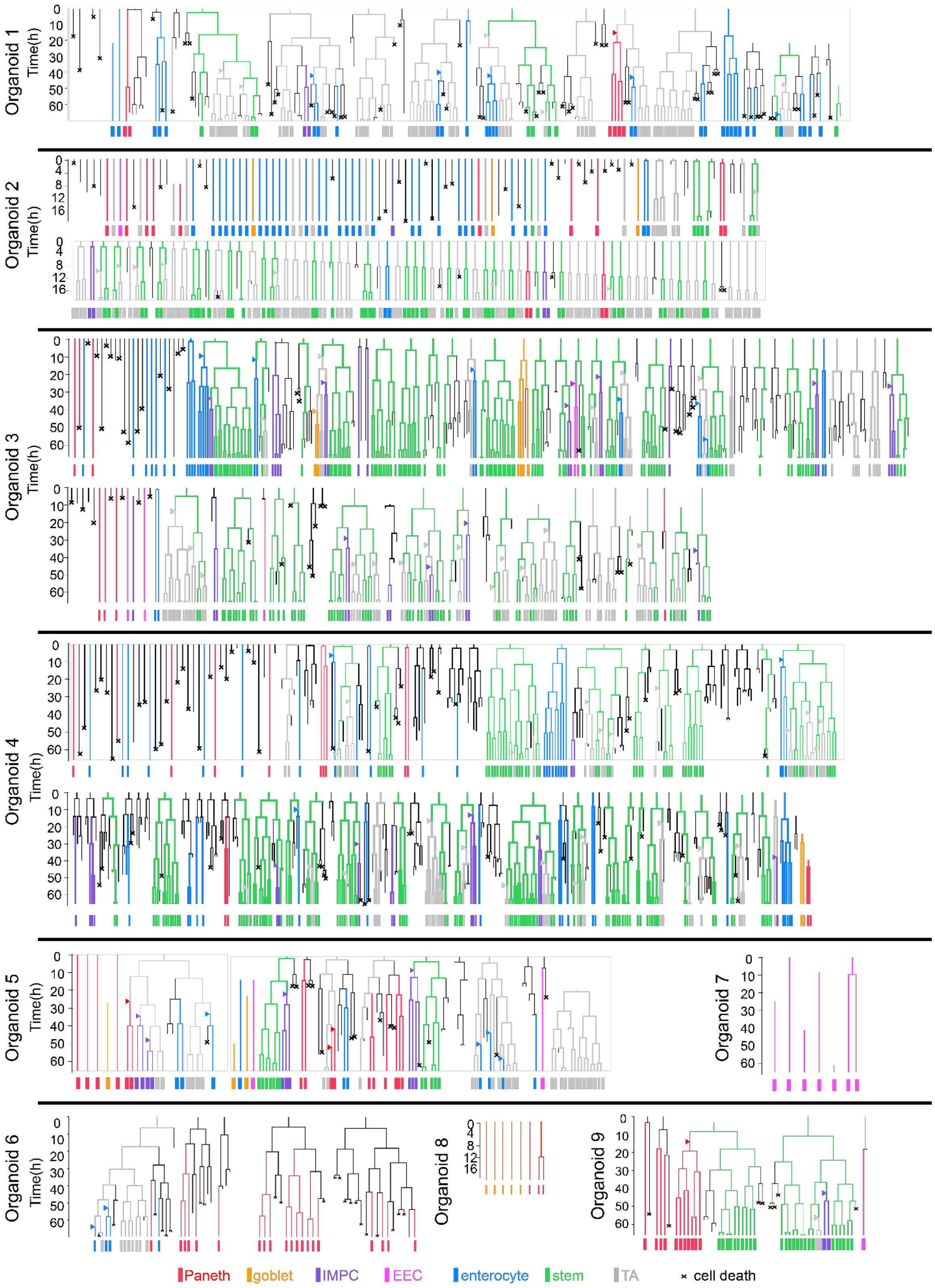

Figure S7. Gallery of lineage trees after backpropagation with inferred cell type transitions. Inferred cell types are shown with different colors. Type transitions are indicated by triangles, colored based on which type the cell was inferred to transition towards. These trees are from nine different organoids.

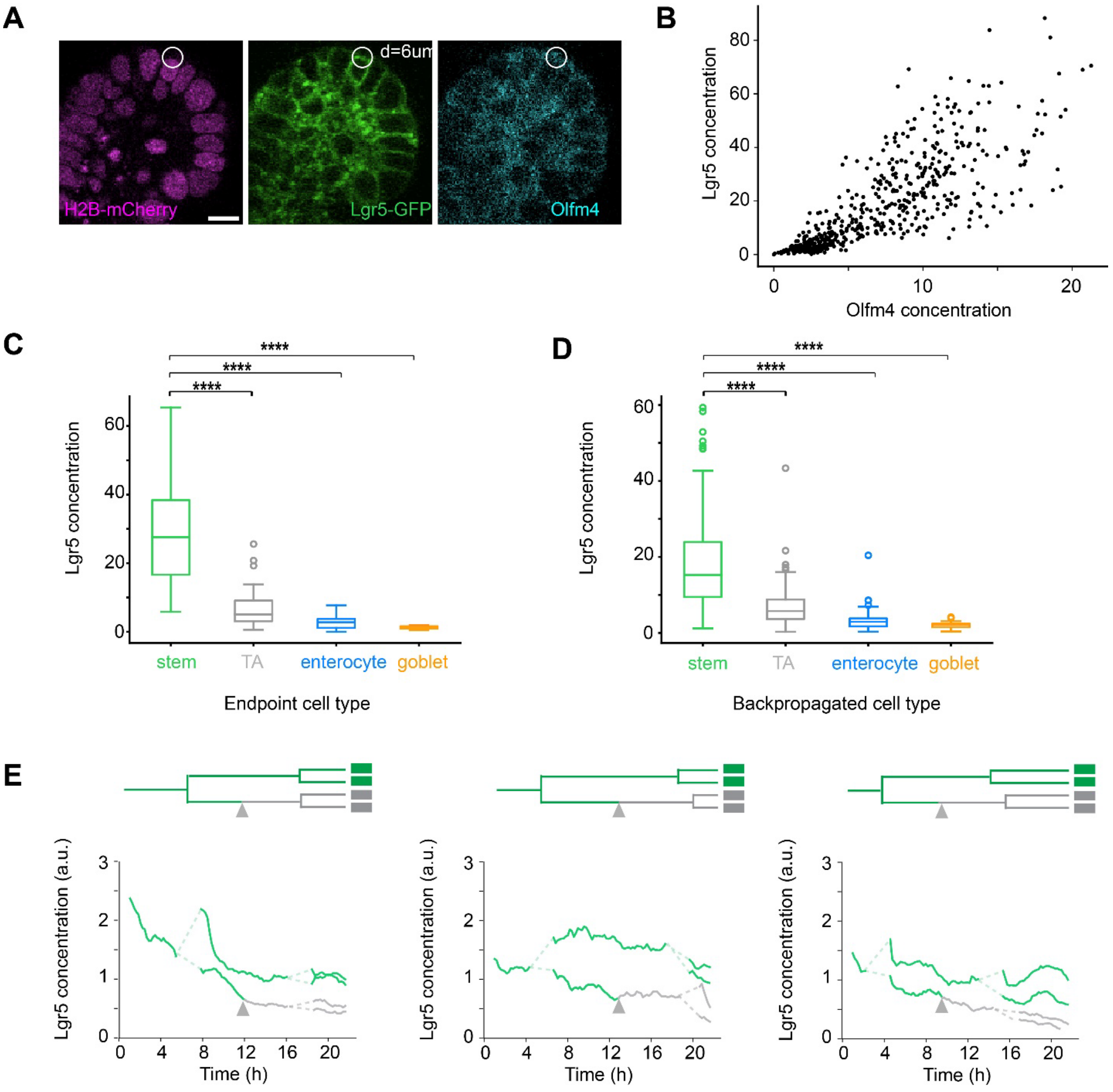

Figure S8. Imaging and tracking with an Lgr5 reporter confirmed the backpropagation method. A, Images of the H2B-mCherry (nuclei), Lgr5-GFP (fluorescent live reporter of stem cells), and Olfm4 (antibody used to identify stem cells). In order to estimate the fluorescence concentration at each cell's membrane, the fluorescence within a 2D circle (d = 6 μm) at the membrane region was measured. **B,** The Lgr5 and Olfm4 fluorescence concentrations, measured by averaging the fluorescence intensity within 2D circles as shown in A, were proportional in single cells. **C,** At the endpoint, the Lgr5 fluorescence concentration of single cells plotted against cell types identified with antibody staining. **D**, The Lgr5 fluorescence plotted against cell type inferred by the TypeTracker method, showing similar distributions to the plots at the endpoint in C. **E**, Lgr5 fluorescence in lineages going through transitions from stem cells to TA cells. Consistently, Lgr5 is lower in lineages that make this transition, compared to the cousin lineages that retain the stem fate. Moreover, the Lgr5 decrease is indeed established in the mother cell that was identified as the committing cell by the TypeTracker method (triangle), thus validating the latter.

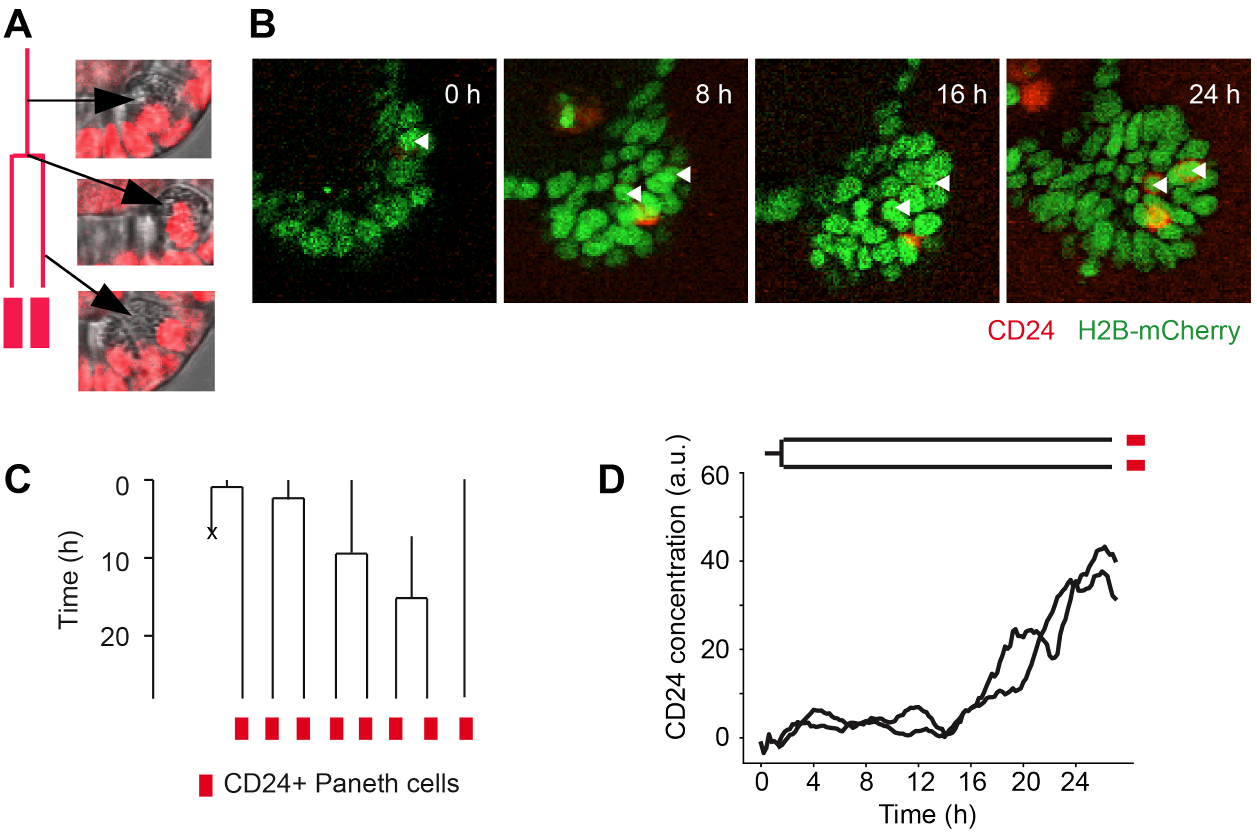

Figure S9. Sister symmetry and real-time marker quantification for Paneth cells. A, Paneth cell divisions. Granules are seen before and after division, evidencing Paneth cell division. **B,** Time-lapse imaging of H2B-mCherry (nuclei) and CD24 (antibody marking Paneth cells). The white arrow indicates a CD24- cell dividing into two cells which both became CD24+ later during imaging. **C,** Lineage trees of CD24+ cells. **D**, The fluorescence signals of CD24 are plotted with time in the example lineage in B.

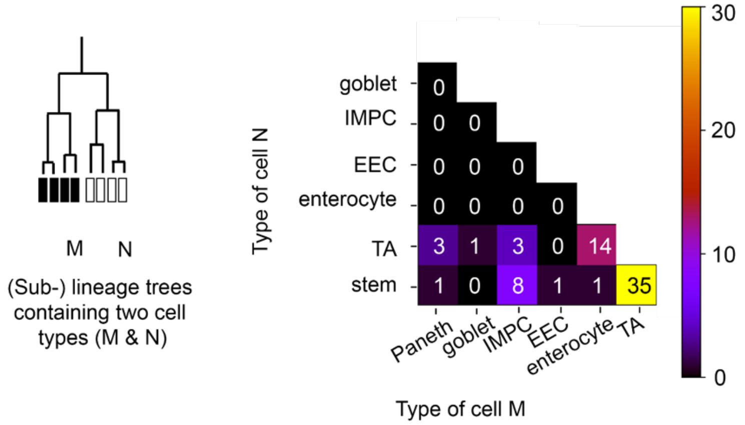

Figure S10. (Sub-) lineage trees containing two cell types often had at least one of the types as either stem cells or TA cells. All the (sub-) lineage trees with two different cell types were taken into account unless more than 50 % of the cells within the lineage could not be tracked or died. The occurrence of each possible combination of the two cell types was counted and shown in the 2D histogram. A combination of two different differentiated cell types, such as enterocytes and goblet cells, was never found. Differentiated cells were often found together with either stem cells or TA cells in the same lineage.

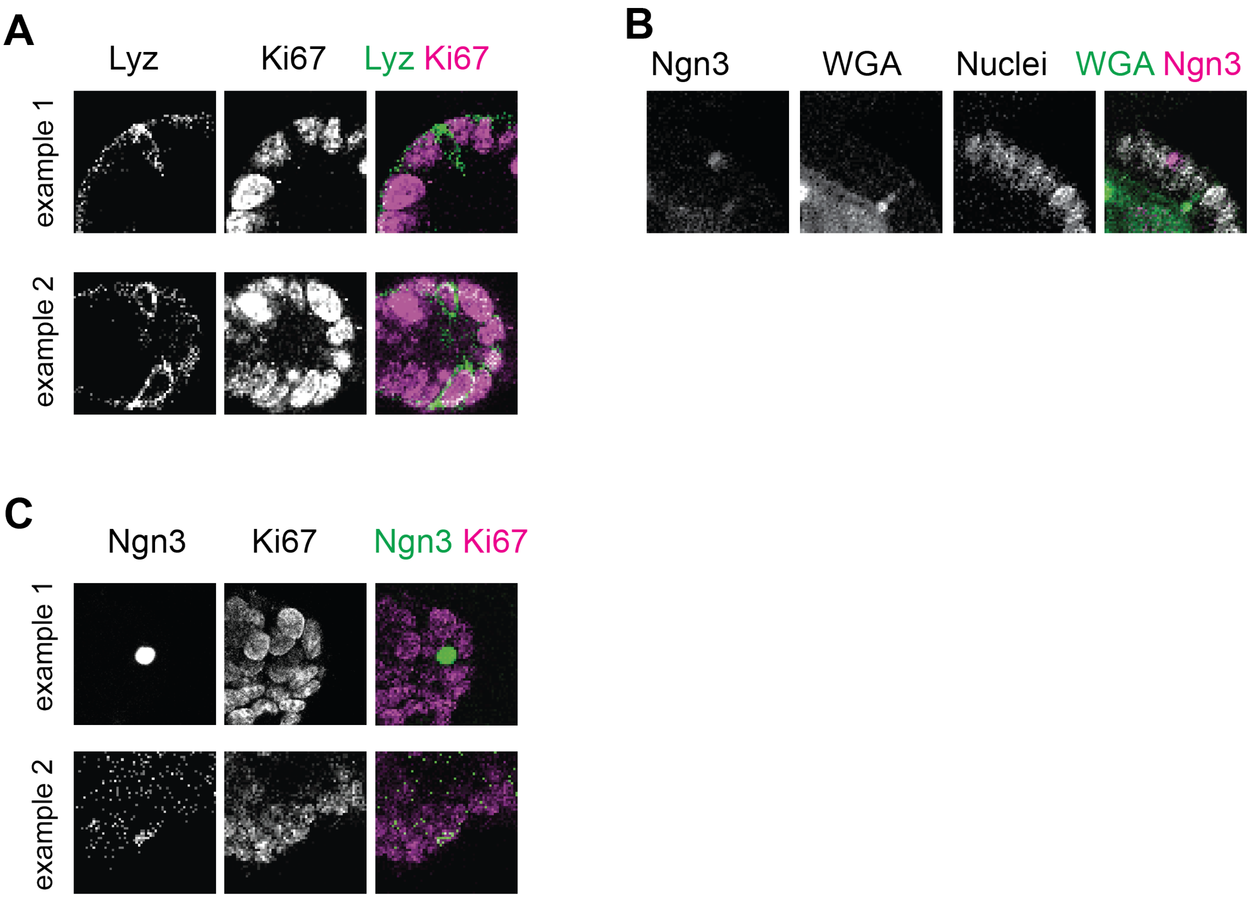

Figure S11. Co-staining cell type markers with Ki67. A, Most Lysozyme (Lyz) positive cells do not co-stain with the proliferation marker Ki67 (example 1, 69%, N = 105 cells), but some do (example 2, 31%, N = 105 cells), supporting the idea that Paneth cells can divide after commitment. **B,** Neurogenin 3 (Ngn3) positive cells do not co-stain with Wheat Germ Agglutinin (WGA, N = 16 cells). **C,** Most Ngn3 positive cells do not co-stain with Ki67 (example 1, 81%, N = 16 cells), but some do (example 2, 19%, N = 16 cells), supporting the idea that Enteroendocrine cells can divide after commitment.

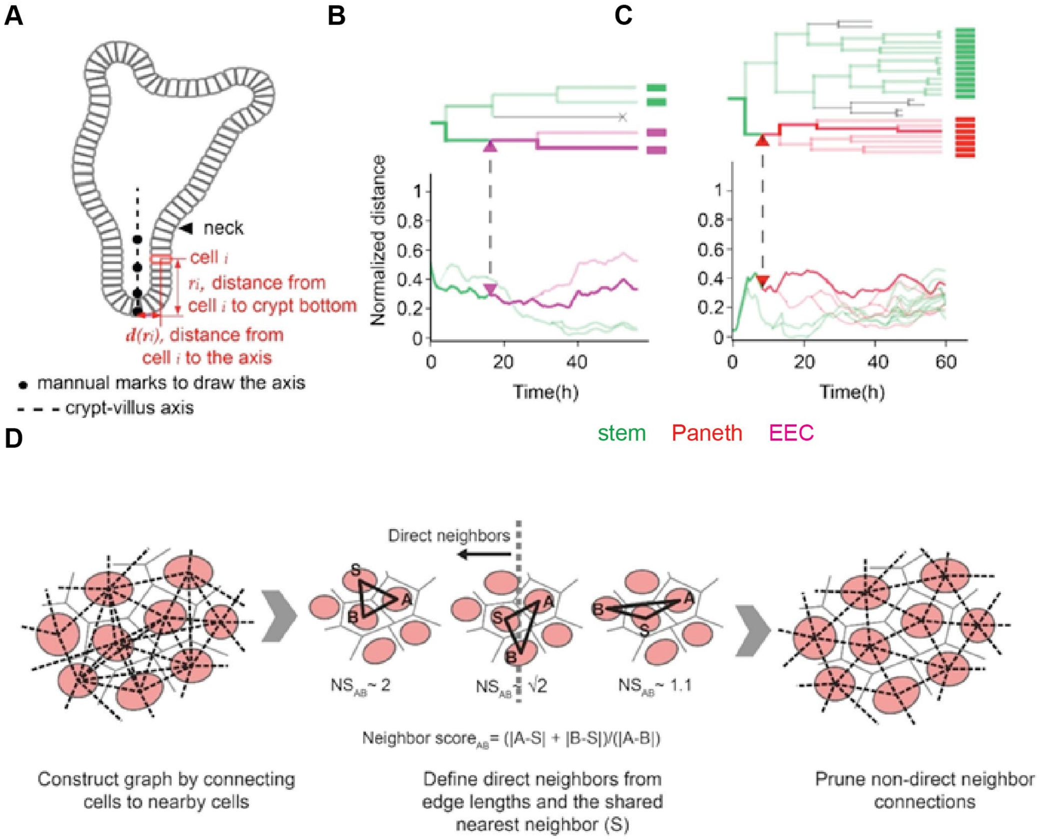

Figure S12. Following the spatial organization of cells during differentiation. A, The crypt-villus axis could be generated by interpolating through the manually annotated points. For each tracked cell i, we determined its position along the axis by finding the value of r_i that minimized the distance $d(r_i)$ between the cell position and the axis. **B & C,** The moving trajectories of cells within different lineages were colored by inferred cell types. Transitions to EECs and Paneth cells took place deep in the crypt, around 0.4, surrounded by stem cells which were often found from 0 to 0.6 along the axis. **D**, Neighbors were defined as pairs of nuclei without another nucleus in between. Each cell's neighbor score for the twenty closest cells (in Euclidean distance) was calculated at every time point. If the neighbor score were higher than $\sqrt{2}$, cells would be identified as neighbors.

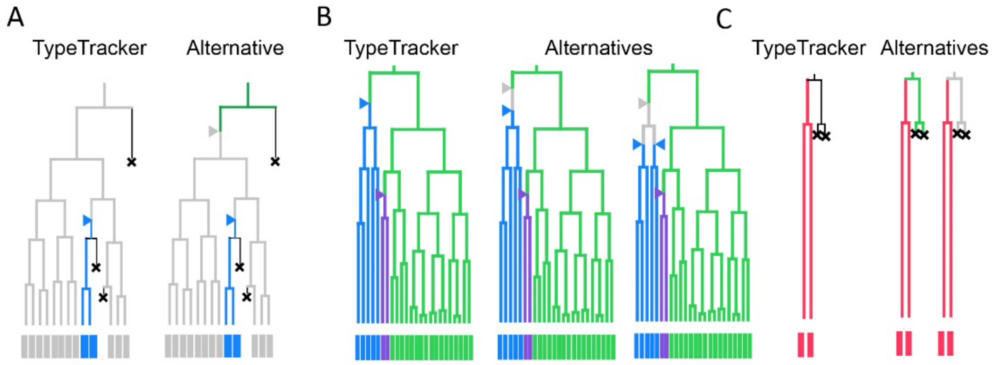

Figure S13. TypeTracker alternative type assignments and potential errors. A, Example lineage tree where the stem and TA fates are ambiguous early in the tree. In this tree, with TA but without stem endpoint fates, the founding cell type may be TA (grey), as TypeTracker would predict, or stem (green) whose descendant lineages are all committed to TA or other types (Alternative). High cell death rates (crosses) exacerbate this issue, as aborted lineages cannot propagate type information back in time. Overall, this problem is mitigated by the fact that proliferative stem and TA types generate multiple branches, which helps their type maintenance in at least one lineage until the end of the tree, and thus proper inference. **B,** Example lineages where a potential TA stage could be missed. **C,** Interrupted lineages. Lineages that are interrupted because of cell death or tracking errors do not provide type information. For instance, in the example shown one then cannot infer the fate of the mother cell that founded the two Paneth cell sisters. This error does not involve an identification ambiguity or error per se, as the lack of information is clear. However, it does limit the backpropagation of genealogically related lineages that are type-identified, as the type of the mother cell then remains undetermined. Such lineages are identified by their black colour in the trees.
